# Immunohistochemical phenotyping of T cells, granulocytes, and phagocytes in the muscle of cancer patients: association with radiologically defined muscle mass and gene expression

**DOI:** 10.1186/s13395-019-0209-y

**Published:** 2019-09-14

**Authors:** Ana Anoveros-Barrera, Amritpal S. Bhullar, Cynthia Stretch, Abha R. Dunichand-Hoedl, Karen J. B. Martins, Aja Rieger, David Bigam, Todd McMullen, Oliver F. Bathe, Charles T. Putman, Catherine J. Field, Vickie E. Baracos, Vera C. Mazurak

**Affiliations:** 1 Department of Agricultural, Food & Nutritional Science, Faculty of Agricultural, Life and Environmental Sciences, University of Alberta, 4-002 Li Ka Shing Centre, Edmonton, Alberta T6G 2P5 Canada; 2 Department of Oncology, University of Calgary, Calgary, Alberta Canada; 3 Flow Cytometry Facility, Faculty of Medicine and Dentistry, University of Alberta, Edmonton, Alberta Canada; 4 Department of Surgery, Faculty of Medicine and Dentistry, University of Alberta, Edmonton, Alberta Canada; 5 Department of Oncology and Department of Surgery, University of Calgary, Calgary, Alberta Canada; 6 Faculty of Kinesiology, Sport, and Recreation, Faculty of Medicine and Dentistry, University of Alberta, Edmonton, Alberta Canada; 7 Department of Oncology, Faculty of Medicine and Dentistry, University of Alberta, Edmonton, Alberta Canada

**Keywords:** Muscle biopsy, Muscle mass, Muscle catabolism, Computed tomography, T cells, CD8 T cells, Granulocytes, Phagocytes, Innate immunity, Adaptive immunity, Cancer

## Abstract

**Background:**

Inflammation is a recognized contributor to muscle wasting. Research in injury and myopathy suggests that interactions between the skeletal muscle and immune cells confer a pro-inflammatory environment that influences muscle loss through several mechanisms; however, this has not been explored in the cancer setting. This study investigated the local immune environment of the muscle by identifying the phenotype of immune cell populations in the muscle and their relationship to muscle mass in cancer patients.

**Methods:**

Intraoperative muscle biopsies were collected from cancer patients (*n* = 30, 91% gastrointestinal malignancies). Muscle mass was assessed histologically (muscle fiber cross-sectional area, CSA; μm^2^) and radiologically (lumbar skeletal muscle index, SMI; cm^2^/m^2^ by computed tomography, CT). T cells (CD4 and CD8) and granulocytes/phagocytes (CD11b, CD14, and CD15) were assessed by immunohistochemistry. Microarray analysis was conducted in the muscle of a second cancer patient cohort.

**Results:**

T cells (CD3+), granulocytes/phagocytes (CD11b+), and CD3−CD4+ cells were identified. Muscle fiber CSA (μm^2^) was positively correlated (Spearman’s *r* = > 0.45; *p* = < 0.05) with the total number of T cells, CD4, and CD8 T cells and granulocytes/phagocytes. In addition, patients with the smallest SMI exhibited fewer CD8 T cells within their muscle. Consistent with this, further exploration with gene correlation analyses suggests that the presence of CD8 T cells is negatively associated (Pearson’s *r* = ≥ 0.5; *p* = <0.0001) with key genes within muscle catabolic pathways for signaling (ACVR2B), ubiquitin proteasome (FOXO4, TRIM63, FBXO32, MUL1, UBC, UBB, UBE2L3), and apoptosis/autophagy (CASP8, BECN1, ATG13, SIVA1).

**Conclusion:**

The skeletal muscle immune environment of cancer patients is comprised of immune cell populations from the adaptive and innate immunity. Correlations of T cells, granulocyte/phagocytes, and CD3−CD4+ cells with muscle mass measurements indicate a positive relationship between immune cell numbers and muscle mass status in cancer patients. Further exploration with gene correlation analyses suggests that the presence of CD8 T cells is negatively correlated with components of muscle catabolism.

**Electronic supplementary material:**

The online version of this article (10.1186/s13395-019-0209-y) contains supplementary material, which is available to authorized users.

## Background

Systemic inflammation is a recognized contributor to muscle wasting in cancer. Pro-inflammatory mediators secreted by the tumor microenvironment and host responsive tissues (i.e., adipose tissue) can activate circulating and tissue-resident immune cells [1]. In the skeletal muscle tissue, interactions between the muscle and immune cells can confer an inflammatory environment promoting muscle loss through various mechanisms. For instance, pro-inflammatory cytokines such as tumor necrosis factor (TNF) alpha and interleukin (IL)-6, secreted by innate (e.g., phagocytes and granulocytes) and adaptive (e.g., T cells) immune cells, can stimulate muscle catabolism by activating nuclear factor kappa-B (NFĸB) [1, 2]. Studies in experimental models report that these inflammatory cytokines can also inhibit anabolic hormones resulting in the impairment of protein synthesis [3]. The integrity of muscle fibers can be compromised by the direct cytotoxic effect of T cells, as observed in muscle inflammatory diseases (e.g., polymyositis and dermatomyositis) in humans [4, 5].

The immune cell presence has been identified with CD45, a general leukocyte marker, in the muscle of patients with early-stage colorectal cancer [6]; however, further characterization and quantification has never been performed in the cancer population. Innate immune cells profiled as neutrophils and macrophages with single immune markers such as CD11b or CD15 or CD14 have been identified in the muscle tissue of populations with myopathy (polymyositis and dermatomyositis), obesity, and exercise-induce stress [7–10]. The presence of T cells within the muscle tissue is commonly explored using CD3 with or without CD4 or CD8 in patients with myopathy and healthy adults undergoing eccentric exercise [7–9, 11, 12].

Exploration of the skeletal muscle immune environment is emerging in human muscle biopsies, which will enable a better understanding of the immune components that contribute to cancer-associated muscle wasting. Also, secondary analysis of oncologic images has enabled the precise characterization of muscle mass distribution in the cancer populations from which these biopsies are derived [13]. Comparison of cancer patients with and without muscle wasting is providing new mechanistic insights into muscle loss [14, 15].

Therefore, in this study, we aimed to investigate the skeletal muscle immune-environment by exploring the presence of different immune cell populations and their relationship to muscle mass in cancer patients undergoing abdominal surgery. It was hypothesized that cancer patients with lower muscle mass have more phagocytes/granulocytes (CD11b, CD15, and CD14) and T cells (CD3, CD4, and CD8) within their muscle tissue.

## Methods

### Study population

Thirty cancer patients undergoing elective abdominal surgery for purposes of cancer diagnosis, primary treatment, or liver metastasis removal were consecutively approached to participate. Previous history of muscle inflammatory disease (i.e., polymyositis, dermatomyositis, and inclusion body myositis) was considered as exclusion criteria. Subject demographics and clinical information were extracted from clinical charts. The majority of patients included in the study were in naïve to chemotherapy and radiotherapy (*n* = 23). Three patients had chemotherapy 9 months prior to the biopsy. Three patients had chemotherapy 1 month prior to the biopsy. One patient received chemotherapy and radiotherapy 4 months prior to the biopsy.

### Radiological assessment of muscle mass by CT scan

**Fig. 1:**
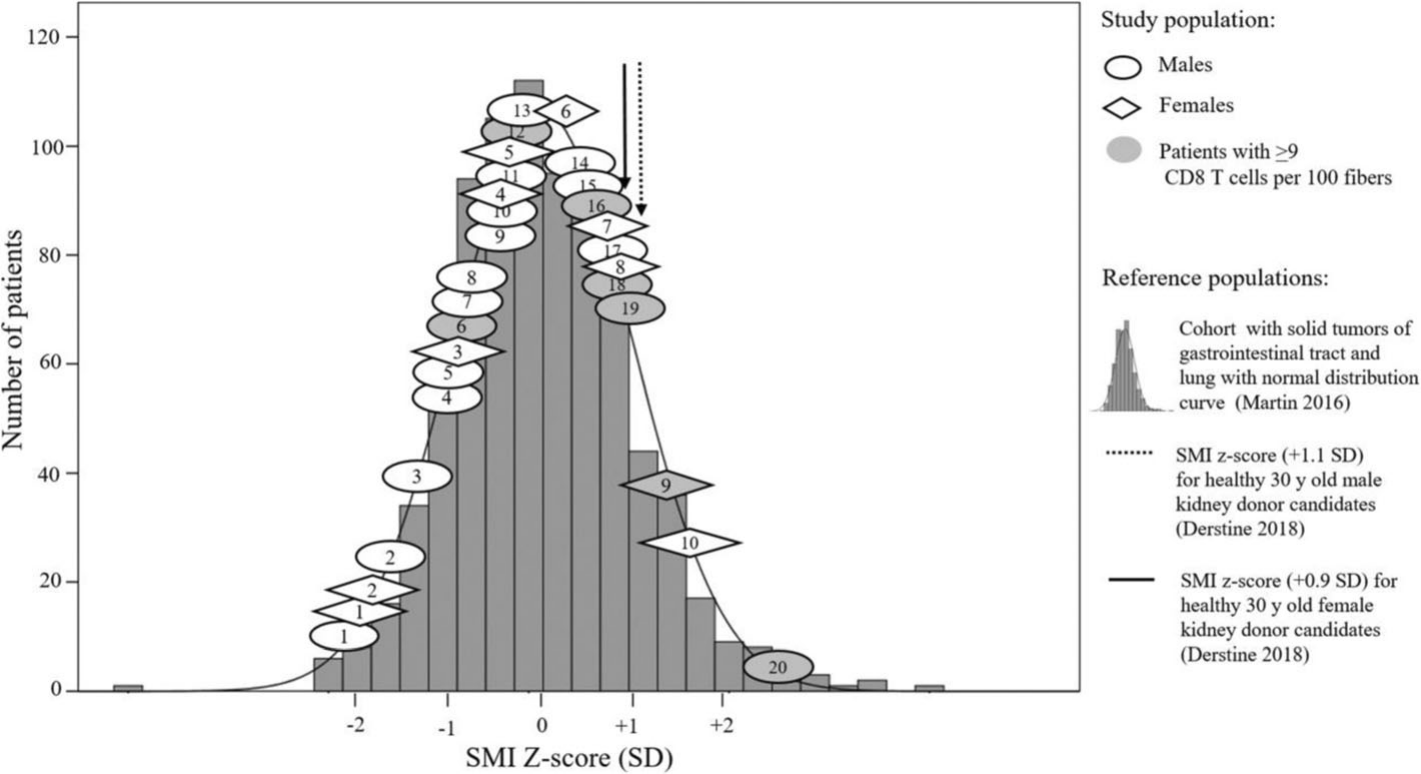
SMI *z*-scores (SD, standard deviation) for male (*n* = 20, circles) and female (*n* = 10, diamonds) patients of the current study in relation to the SMI *z*-score distribution (dark gray histogram) from a cancer cohort with gastrointestinal tract and lung solid tumors (*n* = 1473, 58% males) from the same regional cancer center [13]. SMI *z*-scores were calculated based on sex and age [15]. Circle and diamonds in light gray are patients with highest values of CD8 T cells per 100 fibers. Vertical arrows representing SMI *z*-scores (SD) for healthy 30-year-old kidney donor candidates were placed to highlight cancer patient with SMI *z*-score values that are similar to the healthy population [61]. Notes: Only one SMI distribution is shown indistinctively of sex, as male (*n* = 828) and female (*n* = 645) SMI distribution shapes were the same in the Martin et al. cohort [13]. SMI *z*-scores for healthy kidney donor candidates were calculated by subtracting Martin et al. oncological cohort mean SMI values (males: 51.5 cm^2^/m^2^/females: 41.3 cm^2^/m^2^) from Derstine et al. healthy 30-year-old mean SMI values (males: 60.9 cm^2^/m^2^/females: 47.5 cm^2^/m^2^) and divided by the SD of Martin et al. oncological cohort (males: ± 8.9/females: ± 7)

Pre-operative CT scans performed as the standard of care in the cancer were used to quantify skeletal muscle L3-CSA [16, 17]. Briefly, CT images collected at the 3rd lumbar vertebrae (L3) were analyzed with Slice-O-Matic® V4.3 (Tomovision, Montreal, QB, Canada), L3 muscle CSA was identified at a range of − 29 to + 150 Hounsfield units (HU). Skeletal muscle index (SMI) was obtained by adjusting muscle CSA for height (cm^2^/m^2^). These pre-operative CT images document the inclusion of male and female patients representing the entire SMI distribution of the population being sampled (*n* = 1473) from our regional cancer center in Alberta, Canada (Fig. 1) [18]. SMI *z*-scores for each subject were calculated based on sex and decade of age using mean values and standard deviation of the population cohort [19]. Further group comparison (low and normal muscle mass) was done based in SMI mean ± standard deviation of 30-year-old healthy individuals [20].

### Muscle biopsies

*Rectus abdominis* (≈1 g) was collected at the initial stage of the surgical procedure. An upper abdominal transverse incision was performed, the muscle was collected by sharp dissection without the use of electrocautery, and biopsies were placed on ice within 10 min. On average, a period of 30 min occurred between biopsy removal and arrival in the laboratory. Visually evident adipose and connective tissue was removed from the muscle specimen. For morphological assessment, the tissue was frozen in cooled isopentane and stored at − 80 °C. Sample processing time after the arrival of the specimen to the laboratory was within 1.5 h; procedures were performed under sterile conditions and tissue was kept on ice.

### Immunohistochemistry

Immunofluorescence was performed in transverse serial sections of 10-μm thickness cut with cryostat Leica model CM300 at − 22 °C. Experiments were done using three serial sections, two slides for immune cell identification [antibody combination: (1) CD3, CD4, and nuclear stain and (2) CD11b, CD14, CD15, and nuclear stain] and one slide for muscle fiber area assessment [antibody combination: (3) laminin/dystrophin]. Tissue slides (Apex™ superior adhesive slides, Leica Biosystems) were fixed in acetone at − 20 °C, washed several times in phosphate-buffered saline (PBS), and incubated with blocking solution (PBS-Tween 20, 10% normal goat serum and 1% bovine serum albumin) for 1 h. Sections were washed in PBS prior to incubation with primary antibodies (Additional file 1: Table S1) at 4 °C overnight. Tissue was washed one time in PBS-Tween 20 and six times in PBS before application of secondary antibodies. Secondary antibodies (see Additional file 2: Table S2) used with CD3, CD11b, and laminin/dystrophin was Alexa Fluor® 647 of goat anti-rabbit IgG, with CD4 and CD14 was Alexa Fluor® 568 of goat anti-mouse IgG1, and with CD15 was Alexa Fluor® 488 of goat anti-mouse IgM. After 2 h of secondary incubation at room temperature, sections were washed six times in PBS. Nuclear stain, 4′,6-diamidino-2-phenylindole (DAPI), was added for 2 min. Slides were mounted in ProLong® Diamond Antifade medium, covered with 1.5-thick coverslips and let to dry flat for 12 h.

### Confocal microscopy and histological analysis

Muscle sections were visualized with a spinning disk confocal microscope (Quorum Wave FX Spinning Disc Confocal System – Quorum technologies). *Z*-stacked images taken across a tissue section using the ×20/0.85 oil lens were assembled together and plane-merged to create a composite image to enable the visualization of a whole and clear tissue cross-section. Volocity 6.3 software [PerkinElmer, Waltham, MA, USA] was used to capture, merge, and analyze all images.

#### Immune cell quantification

Immune cells located in the endomysium were quantified. Assessments were performed on a total area of 2.0 ± 0.7 mm^2^ which included information of 398 ± 97 muscle fibers. Manual immune cell quantification was performed using Volocity® software, and results were reported as the number of cells per 100 fibers. Values were also presented as the number of cells per mm^2^ for comparison with other study populations reported in the literature. CD3+CD4− cells were profiled as CD8 T cells. Areas excluded for immune cell quantification were large blood vessels and detectable capillaries, nerves, fibrotic tissue, and folded areas. Immune cell populations were identified based on the co-expression of cell surface markers, and immune cells were quantified only if they had a nucleus surrounded by the fluorescent antibody on the surface (see Additional file 3: Figure S1). Endomysial areas and muscle fibers were located by increasing the brightness of the image as muscle tissue autofluorescence easily allowed the identification of muscle structures. In addition, muscle fibers and endomysium location were corroborated using serial sections of laminin-dystrophin stain (see Additional file 4: Figure S2).

#### Muscle fiber CSA analysis

Image assessment of muscle fiber CSA was done using in Volocity® software. First, muscle fiber CSA (μm^2^) was obtained from the detection of membrane fluorescence of single muscle fibers. Second, a mean muscle fiber CSA value was calculated from the area assessment of all muscle fibers per cross section.

#### Immune cell isolation and flow cytometry analysis

Mononuclear cells were isolated from the *rectus abdominis* muscle in a subset of *n* = 17 (males = 12/females = 5) patients using similar procedures to those previously described [21]. Briefly, 150 mg of the muscle was digested in an enzymatic solution (DMEM with collagenase 1%, dispase II, and CaCl_2_) at 37 °C (5% CO_2_). After washing, the sample was filtered through a mesh. The pellet was resuspended in PBS with 4% FBS, and cells counted using trypan blue. Volume equivalent to one million mononuclear cells was used. Conjugated antibodies (CD3−BV421 and CD11b−PE-Cy7; BD Biosciences) for cell surface antigens were mixed in aliquot cocktails and added into the respective tubes, including fluorescence minus one controls (FMOs). Samples were incubated at 4 °C in the dark for 40 min. After incubation, cells were washed twice and fixed with 100 μL of 1% paraformaldehyde. Tubes were refrigerated for 12 h until flow cytometry acquisition was performed with a BD Fortessa X-20 analyzer and BD FacsDiva™ software [BD Biosciences]. Finally, flow cytometry analyses were done using FlowJo© software [FlowJo, LLC] (Additional file 5: Figure S3).

### Gene expression: microarray analysis

Microarray analysis was conducted in the *rectus abdominis* muscle from a cohort of patients (*n* = 69 males/*n* = 64 females) previously described [22]. Briefly, total RNA was isolated, purified, quantified, and subjected to linear amplification and Cy3 labeling and hybridization to Agilent Whole Human Genome Arrays using Agilent kits (One Color Low RNA Input Linear Amplification Kit Plus, One Color RNA Spike-In Kit and Gene Expression Hybridization Kit) according to the manufacturer’s protocols. The arrays were scanned using an Agilent Scanner, the data was extracted, and quality was evaluated using Feature Extraction Software 10.5.1 (Agilent). The data were normalized using GeneSpring GX 11.5.1 (Agilent). The data used in this publication have been deposited in the US National Center for Biotechnology Information (NCBI) Gene Expression Omnibus25 and are accessible through GEO series accession number GSE41726.

### Statistical analysis

All analyses were conducted in IBM® SPSS® Statistics software, version 24. A test for normal distribution was applied to the continuous variables, and immune cells were not normally distributed. To assess associations between immune cells and muscle mass values, Spearman’s coefficient was selected. Comparisons between sexes and groups were conducted using Mann-Whitney *U* (non-categorical variable) and chi-square or Fisher’s exact test (categorical variables) where appropriate. Statistical significance was considered at *p* values less than 0.05.

#### Gene correlation analyses

Pearson correlation analysis was done in R version 3.4.2 (2017-09-28) using the Hmisc (version 4.1-1) and corrplot (version 0.84) packages. Correlation analysis between CD8A and all other genes in the microarray was used to identify significantly correlated genes. The correlation *p* values were adjusted for multiple comparisons using the Benjamini-Hochberg procedure. Correlations were considered significant if they had a false discovery rate (FDR) of < 0.05. Of those genes significantly correlated with CD8A, genes associated with CD8 T cell function and muscle catabolism were identified based on a review of the current literature and submitted to further analysis. Correlations between all gene-gene combinations were conducted to examine the relationship between CD8 T cell genes and catabolic genes.

## Results

Patient characteristics are presented in Table 1. Mean age was 64 ± 11 years, and there were no age differences between men and women. Most patients had gastrointestinal malignancies (91%), with colorectal and pancreatic cancer being common among males and females. Advance stage (IV) was present in 60% of men and 80% of women.

**Table 1:** Patient characteristics

	All (*n* = 30)	Males (*n* = 20)	Females (*n* = 10)	*p*
Age, mean years ± SD (Min-Max)	64 ± 11 (38–81)	63 ± 13 (38–81)	67 ± 7 (52–77)	0.71
Tumor type, % (*n*)				0.68
Colorectal	37 (11)	35 (7)	40 (4)	
Pancreas	30 (9)	25 (5)	40 (4)	
Liver	10 (3)	10 (2)	10 (1)	
Bile duct	7 (2)	10 (2)	0 (0)	
Gallbladder	7 (2)	5 (1)	10 (1)	
Others	9 (3)	15 (3)	0 (0)	
Tumor stage, % (*n*)				0.22
I	3.3 (1)	5 (1)	0 (0)	
II	3.3 (1)	5 (1)	0 (0)	
III	13 (4)	10 (2)	20 (2)	
IV	67 (20)	60 (12)	80 (8)	
N/A	13.3 (4)	20 (4)	0 (0)	
BMI (kg/m^2^), mean ± SD	27 ± 7	27 ± 6	26 ± 9	0.68
BMI classification, % (*n*)				0.24
Underweight	10 (3)	10 (2)	10 (1)	
Normal	47 (14)	40 (8)	60 (6)	
Overweight	23 (7)	30 (6)	10 (1)	
Obesity I	7 (2)	5 (1)	10 (1)	
Obesity II	10 (3)	15 (3)	0 (0)	
Obesity III	3 (1)	0 (0)	10 (1)	
Comorbidities, % (*n*)				
Diabetes type II	30 (9)	20 (4)	50 (5)	0.96
Hypertension	50 (15)	50 (5)	50 (5)	1.00
CVD	23 (7)	25 (5)	20 (2)	0.76
Dyslipidemia	23 (7)	25 (5)	20 (2)	0.76
Computed tomography body composition analysis, mean ± SD				
L3 Muscle CSA (cm^2^)	132.1 ± 33.9	146.8 ± 29.5	102.6 ± 20.3	< 0.01*
SMI (cm^2^/m^2^)	45.8 ± 9.2	49 ± 8.5	40.5 ± 8.4 *	0.39
SMI z-score (SD)	−0.2 ± 1.1	−0.3 ± 1.1	−0.1 ± 1.2	0.59
L3 Muscle radiodensity (HU)	30.8 ± 9.5	31.4 ± 10.5	30.4 ± 7.6	0.72
L3 VAT CSA (cm^2^)	172.3 ± 91.9	188 ± 97.2	150.6 ± 79.7	0.29
L3 SAT CSA (cm^2^)	192.8 ± 115.2	165.2 ± 110.7	247.5 ± 102.2	0.015*
L3 TAT CSA (cm^2^)	377.1 ± 172.3	365.2 ± 173	409.5 ± 168.9	0.45
Muscle histological characteristics: Muscle fiber CSA (μm^2^), mean ± SD	3154 ± 1408	3707 ± 1329	2047 ± 788*	< 0.01*

Others: melanoma, chronic lymphocytic leukemia, and lymphoma. *N/A* no applicable, *BMI* body mass index, *CVD* cardiovascular disease, *L3* lumbar 3, *CSA* cross-sectional area, *HU* Hounsfield unit, *VAT* visceral adipose tissue, *SAT* subcutaneous adipose tissue, *TAT* total adipose tissue, *SMI* skeletal muscle index, *SD* standard deviation. *Difference between males and females (*p* = < 0.05)

### Innate and adaptive immune cells are present in the muscle of cancer patients

**Fig. 2:**
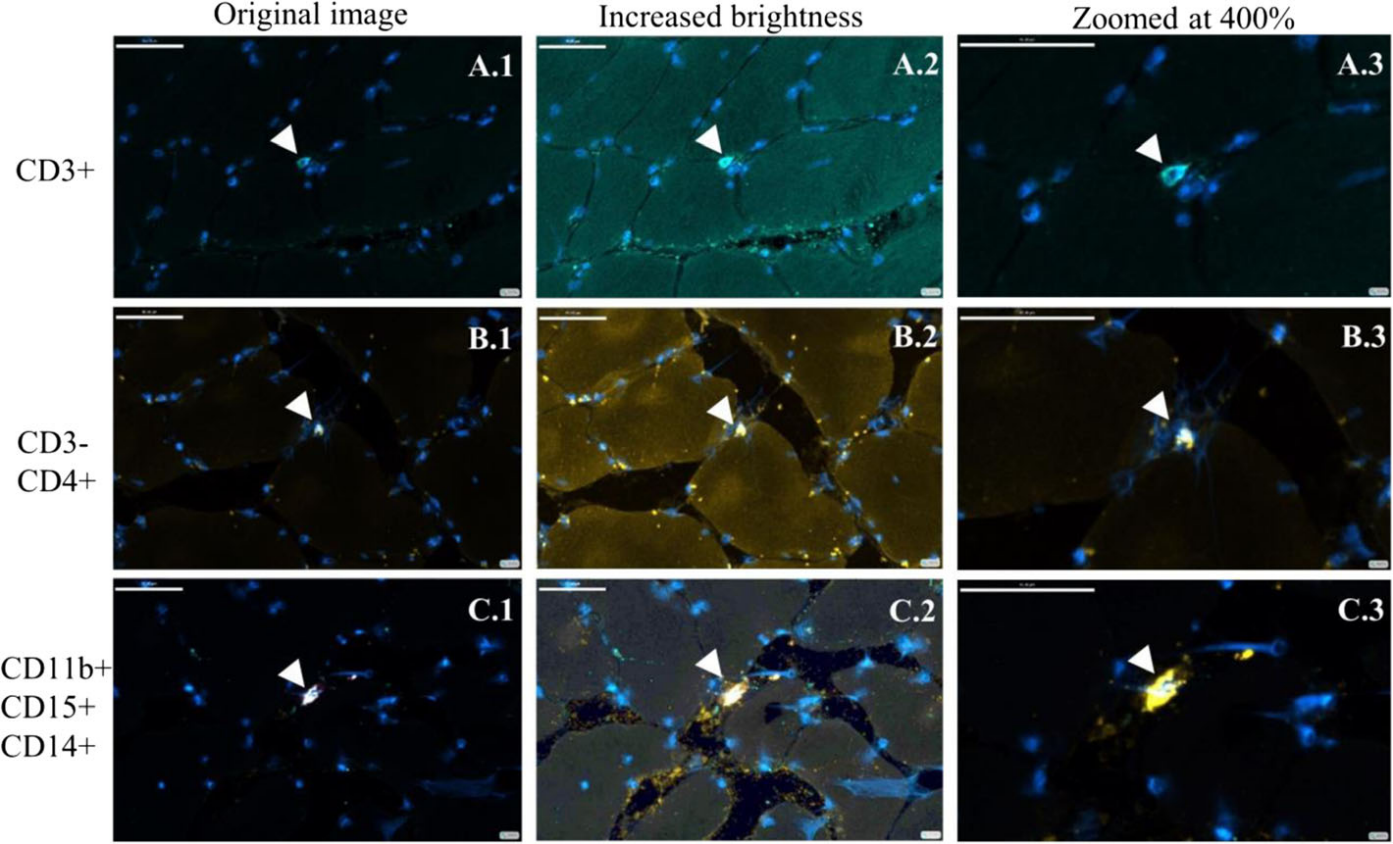
Immunostaining of CD3+ (**A.1–A.3**), CD3−CD4+ (**B.1–B.3**), and CD11b+CD14+CD15+ (**C.1–C.3**) cells pointed by the white arrows in the muscle tissue of cancer patients. Stained nuclei in blue. **A.1**, **B.1**, and **C.1** are the original images with no brightness manipulation. As for images **A.2**, **B.2**, and **C.2**, brightness was increased to visually appreciate the location of the immune cells on the periphery of muscle fibers. **A.3**, **B.3**, and **C.3** were zoomed to a 400% from the original image. Scale bar 45 μm

**Fig. 3:**
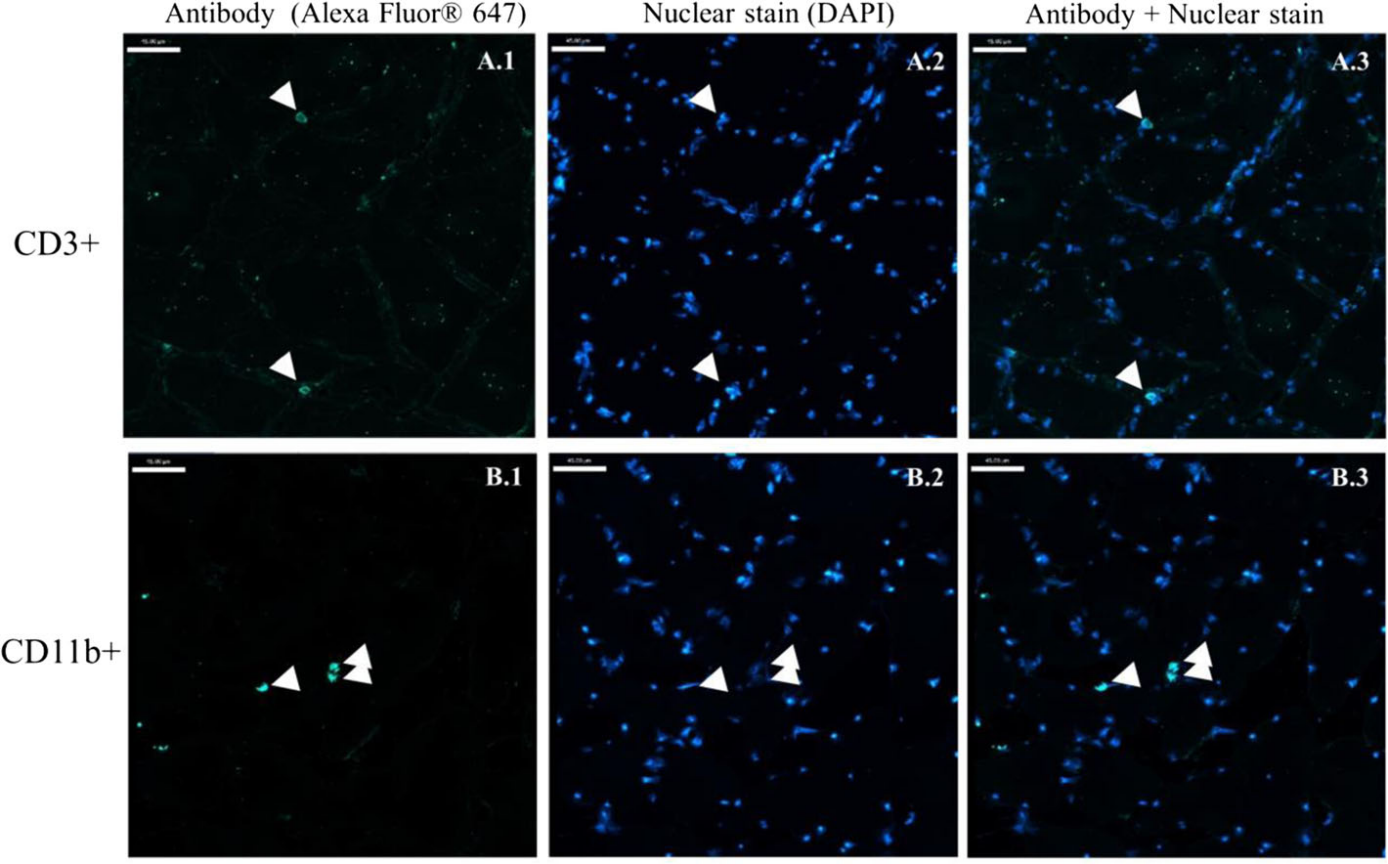
Representation of the distribution of more than one immune cell localized on a muscle cross section. Immunostaining of CD3+ (**A.1–A.3**) and CD11b+ (**B.1–B.3**). **A.1** and **B.1** antibody detected by Alexa Fluor® 647. **A.2** and **B.2** nuclear stain (DAPI). **A.3** and **B.3** antibody and nuclear stain. Scale bar 45 μm

T cells, CD3−CD4+, and granulocytes/phagocytes were identified in the muscle tissue of patients diagnosed with cancer (Table 2 and Fig. 2). Immune cells were consistently found in the periphery of the muscle fibers, in the area known as endomysium (Fig. 2). In general, the distribution of immune cells occurred along the tissue with a clear separation between cells (Fig. 3).

**Table 2:** Immunohistochemical identification and quantification of immune cells in the muscle

Immune cell identified	Antibodies	All (*n* = 30)	Males (*n* = 20)	Females (*n* = 10)	
		No. of cells per 100 fibers			*p* value
Granulocytes/phagocytes	CD11b+	1.6 (0–8)	1.6 (0–6)	1.6 (1–8)	0.98
a. Granulocytes/phagocytes subtype 1	CD11b+CD14+CD15+	1.0 (0–5)	1.3 (0–4)	0.5 (0–5)	0.32
b. Granulocytes/phagocytes subtype 2	CD11b+CD14−CD15−	0.5 (0–3)	0.0 (0–2)	1.1 (0–3)	0.14
T cells	CD3+	6.5 (1–24)	8.3 (1–24)	5.5 (1–12)	0.07
a. CD4 T cells	CD3+CD4+	2.3 (0–15)	2.7 (0–15)	1.7 (0–6)	0.05*
b. CD8 T cells	CD3+CD4−	4.1 (1–18)	5.6 (1–18)	4 (1–10)	0.21
CD3−CD4+ cells	CD3−CD4+	0.6 (0–12)	2.0 (0–6)	0.7 (0–12)	0.71

Values reported as median (range). *p* values from Mann-Whitney *U* test. *Difference between males and females (*p* = < 0.05)

T cells accounted for 67% of immune cells identified within the muscle tissue (Table 2). CD8 T cells (CD3+CD4−) were twice as abundant as CD4 T cells (CD3+CD4+). A population of CD3−CD4+ cells was identified and represented 14% of the immune cells. CD11b+cells comprised 20% of the immune cells identified, and two subtypes of CD11b+cells were identified. CD11b+CD14+CD15+ cells were present at 1.6 fold higher than CD11b+CD14−CD15− cells. Immune cells with single antigens for CD14 or CD15 were not present. Males had more CD4 T cells than females (Table 2). No other differences between men and women were observed.

Flow cytometry analysis enabled confirmation of the immune populations explored in the immunohistochemistry assays (see Additional file 8: Figure S3). Immune cells comprised a very small percentage of isolated mononuclear cells. CD3+ cells comprised of 1.8 ± 1% (mean ± standard deviation) and CD11b+ of 1.6 ± 1.3% (mean ± standard deviation). No statistical differences were found between males and females for CD3 (*p* = 0.2) or CD11b cells (*p* = 0.9). No further analyses were pursued given the low numbers of CD3+ and CD11b+ cells obtained from the muscle tissue.

#### T cells (CD3+), CD3−CD4+ cells, and granulocytes/phagocytes (CD11b+) cells are positively correlated with muscle mass

Muscle mass was evaluated by histological and radiological assessments (Table 1). Muscle mass measurements are reported as muscle fiber CSA, L3-CSA, SMI, and SMI *z*-scores. Correlations between immune cells and muscle mass variables are reported in Table 3. Muscle fiber CSA was positively correlated with total CD3+, CD4 (CD3+CD4+) and CD8 (CD3+CD4−) T cells, CD3−CD4+ cells, and granulocytes/phagocytes subtype 1 (CD11b+CD14+CD15+). SMI and SMI *z*-scores were positively correlated with total CD3+ T cells and CD8 T cells.

**Table 3:** Correlation between the number of immune cells and muscle mass variables, all patients (*n* = 30)

		Total T cells	CD4 T cells	CD8 T cells	CD3−CD4+ cells	Total granulocytes/phagocytes	Granulocytes/phagocytes subtype 1	Granulocytes/phagocytes subtype 2
Muscle fiber CSA	*r*	0.63	0.41	0.63	0.45	0.29	0.46	− 0.05
	*p*	*0.0002*	*0.026*	*0.0002*	*0.012*	0.126	*0.011*	0.784
SMI	*r*	0.49	0.28	0.44	0.2	− 0.003	0.14	− 0.16
	*p*	*0.006*	*0.136*	*0.014*	0.59	0.986	0.44	0.395
SMI *z*-scores	*r*	0.44	0.12	0.49	0.24	− 0.04	0.05	− 0.12
	*p*	*0.016*	*0.517*	*0.005*	0.186	0.823	0.782	0.527

*r* = Spearman’s coefficient of correlation. *p* = < 0.05: statistical significance. *CSA* cross-sectional area, *SMI* skeletal muscle index

In women, significant correlations between muscle fiber CSA, CD3+ T cells (*r* = 0.8, *p* = 0.006), and CD8 (CD3+CD4−) T cells (*r* = 0.9, *p* = 0.001) were observed. In men, muscle fiber CSA was positively correlated with total number of CD11b+ cells (*r* = 0.5, *p* = 0.01), CD11b+CD14+CD15+ (*r* = 0.6, *p* = 0.007), total CD3+ T cells (*r* = 0.5, *p* = 0.01), and CD8 (CD3+CD4−) T cells (*r* = 0.6, *p* = 0.01). In addition, CD3+ T cells were correlated with SMI (*r* = 0.6, *p* = 0.006) and SMI *z*-scores (*r* = 0.6, *p* = 0.004); CD8 (CD3+CD4−) T cells to SMI (*r* = 0.5, *p* = 0.004) and SMI *z*-scores (*r* = 0.6, *p* = 0.005) in men.

### Comparison of patient characteristics based on normal and low muscle mass

Cancer patients participating in the present study represent the entire SMI distribution reported in a population from the same region with solid gastrointestinal and respiratory tumors (Fig. 1). A subset of patients (37%) was identified as having SMI values within what is reported for normal muscle mass in 30-year-old individuals (Table 4) [20]. Further comparison of patient characteristics between normal and low muscle mass revealed no differences in age and number of men between groups. The prevalence of patients with stage IV and chemotherapy was higher in the low muscle mass group. Patients with the highest proportions of CD8 T cells (> 9 cells per 100 fibers) were present in the normal muscle mass group (Table 4 and Fig. 1).

**Table 4:** Comparison of characteristics of cancer patients with low muscle mass and normal muscle mass

	Normal muscle mass	Low muscle mass	*p* value
Total sample size, *n*	11	19	
Males, % (*n*)	55 (6)	74 (14)	0.25
Age, mean years ± SD	63.5 ± 13	64.3 ± 10.4	0.89
SMI (cm^2^/m^2^), mean ± SD			
Males	59 ± 4.9	44.6 ± 5.4	< 0.01
Females	47.2 ± 4.0	33.8 ± 5.7	< 0.01
Patients with stage IV, % (*n*)	55 (6)	74 (14)	0.25
Patients exposed to chemotherapy, % (*n*)	0 (0)	37 (7)	0.025
Patients with > 9 CD8 T cells per 100 fibers, % (*n*)	45 (5)	11 (2)	0.043

Normal muscle mass: patients with SMI (cm^2^/m^2^) within the mean ± standard deviation (SD) of 30-year-old healthy individuals (males: 60.9 ± 7.8 cm^2^/m^2^//females: 47.5 + 6.6 cm^2^/m^2^) as reported by Derstine et al. [20] Differences between groups were analyzed by Mann-Whitney *U* test (non-categorical variables) and Fisher’s exact test (categorical variables) where appropriate. Statistical significance *p* < 0.05

### Gene analysis: CD8 T cells negatively associated with catabolic pathways in the muscle

**Fig. 4:**
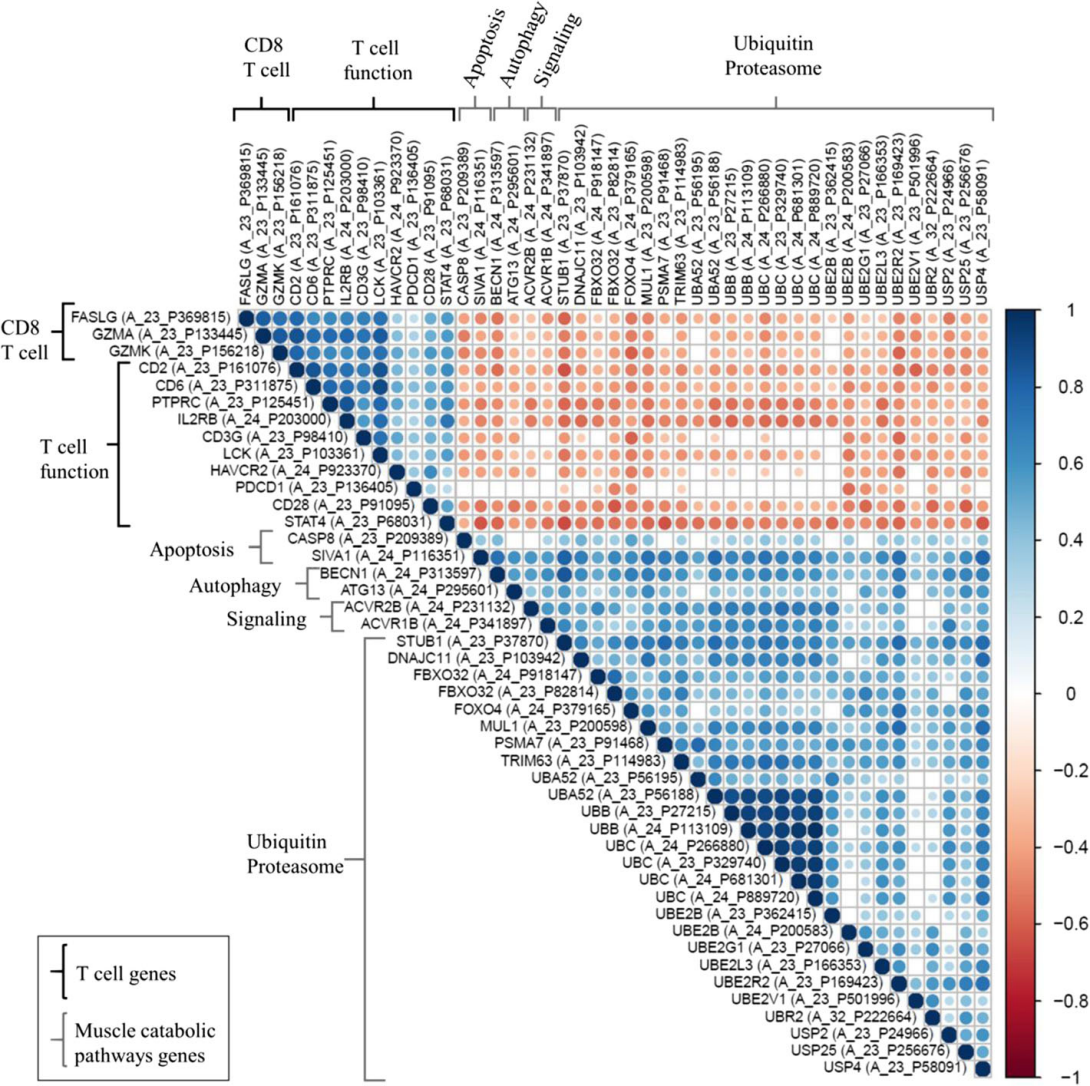
Correlation matrix of T cells genes and muscle catabolic pathway genes. The strength of the correlation is represented by the size and color intensity of each spot, positive in blue and negative in red. Pearson’s correlation analysis. Gene arrays from the *rectus abdominis* muscle from the secondary male cohort (*n* = 69)

Microarray analyses were performed in the *rectus abdominis* muscle of a second cohort with similar clinical characteristics to our main cohort (see Additional file 6: Table S3) [22]. Forty-six genes were selected for this analysis representing T cells and CD8 T cell function [23–28], as well as muscle catabolic pathways (Additional files 7 and 8: Table S4 and Table S5) [1, 2, 29–38]. The gene correlation matrix is presented in Fig. 4 for men (see Additional file 9: Figure S4 for women). CD8 T cell-associated genes and muscle catabolic genes were inversely correlated in both men and women (Additional files 7 and 8: Table S4 and Table S5); however, more consistent and stronger correlation coefficients were observed in men in comparison to women, as we had seen for the immune cell counts and measures of muscle mass. In men, T cell function and CD8 T cell-specific genes were negatively correlated with ubiquitin-proteasome genes (FOXO4, STUB1, UBE2R2, and USP2) and autophagy/apoptosis genes (BECN1). Genes from the ubiquitin proteasome (TRIM63, MUL1, FBXO32, UBB, UBC, USP4, UBE2B, UBE2L3, UBA52, and DNAJC11), catabolic signaling (ACVR2B and ACVR1B), apoptosis (SIVA1), and autophagy (ATG13) pathways were negatively correlated with T cell function genes. Apoptotic gene CASP8 was negatively correlated with CD8 T cell-specific gene GZMA.

Further gene exploration on the correlation of CD8A gene with regulatory cytokine IL-10 and pro-inflammatory cytokine IL-6 was done. A significant positive correlation was found between CD8A and IL-10 receptor subunit *α* (*r* = 0.6, *p* = < 0.001), and no significant correlations were observed for IL-10 (*r* = 0.2, *p* = 0.2), IL-6 (*r* = 0.2, *p* = 0.19), or IL-6 receptor (*r* = − 0.2, *p* = 0.07).

## Discussion

This is the first study to investigate the local immune environment of the skeletal muscle by identifying immune cell populations in the muscle and their relationship to muscle mass in cancer patients. T cells, CD3−CD4+ cells, and granulocytes/phagocytes were identified. Assessment of the relationship between immune cell numbers and muscle mass revealed a greater number of T cells (CD3+), granulocyte/phagocytes (CD11b+CD14+CD15+) and CD3−CD4+ cells in patients with larger muscle fiber size. In particular, CD3+CD4− cells, profiled as CD8 T cells by exclusion, were positively correlated with both histological and radiological indices of muscle mass. In support of this finding, CD8 (CD3+CD4−) T cell-associated genes were negatively correlated with genes involved in muscle catabolic pathways.

**Fig. 5:**
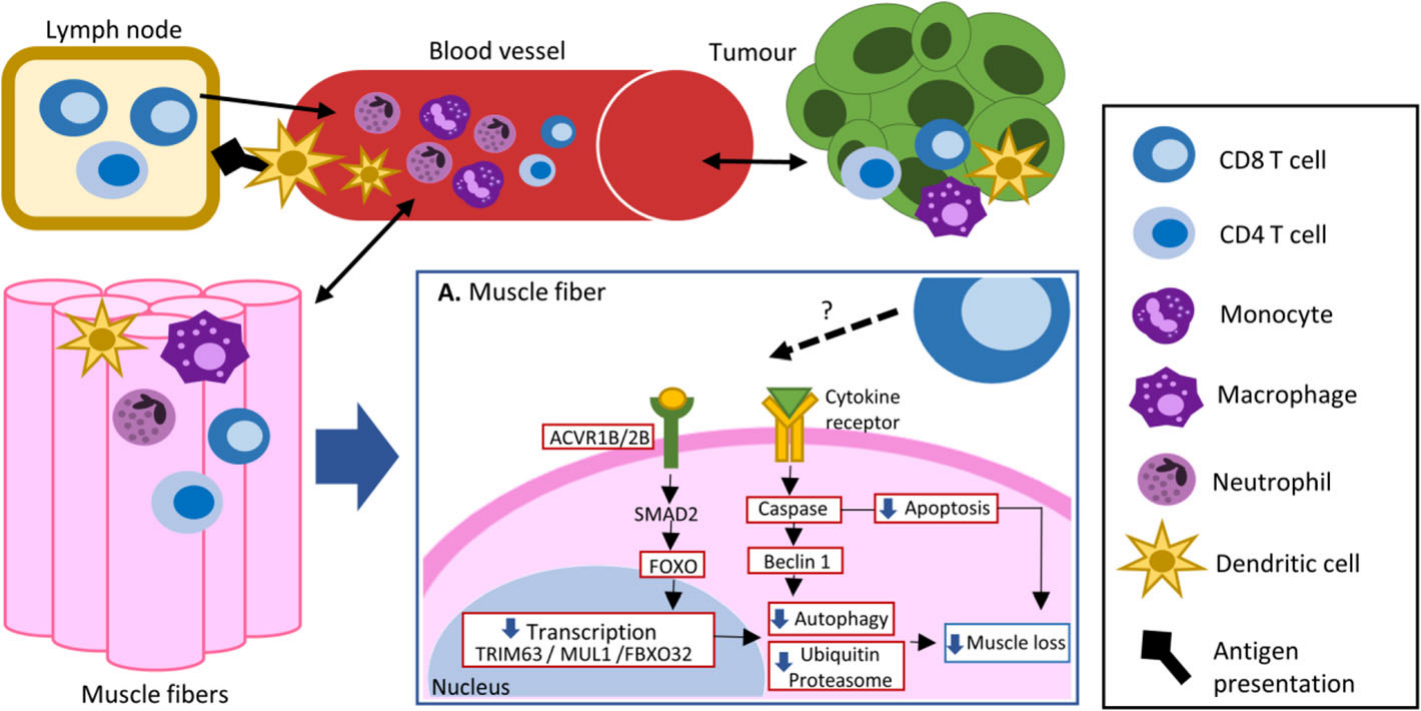
The presence of immune cells in the skeletal muscle tissue during cancer and the potential role of CD8 T cells in the muscle mass preservation. During cancer, changes in immune cell populations occur. Inflammatory mediators secreted by the tumor are capable of activating and mobilizing circulating and tissue-resident immune cells. The presence of T cells (CD8 and CD4) within the muscle tissue occurs in collaboration of antigen-presenting cells (i.e., dendritic cells) that travel from the tissues into the bloodstream and lymph nodes. Once in the muscle, cytokine secretion by T cells, granulocytes (i.e., neutrophils), and phagocytes (i.e., macrophages and dendritic cells) promote further recruitment and phenotype polarization (inflammatory or anti-inflammatory) of immune cells. A. Close-up of the muscle fiber and nucleus. Gene correlation analysis suggests an inverse relationship of CD8 T cells with diverse components (in red boxes) of muscle catabolic pathways which might impact muscle mass

In cancer preclinical models, mobilization of immune cells participating in innate and adaptive responses has been observed in several tissues and organs (i.e., blood, brain, liver, adipose tissue) [39]. It is suggested that immune mediators originating in the tumor micro-environment could influence the rearrangement of the immune environment of the skeletal muscle influencing muscle mass (Fig. 5) [40–44]. In humans, linear relationships between blood immune cell counts, used as markers for systemic inflammation, and radiological assessments of muscle mass have been reported [45–51]. Similar to our CD3+ T cell findings, peripheral lymphocyte counts, which include T cells and other adaptive responders, have been positively associated with higher SMI in gastrointestinal and lung cancers [48–53]. Elevated levels of circulating granulocytes, in particular, neutrophils, are consistently reported in cancer patients with low muscle mass [54–57], which is contrary to our results from CD11b+ phagocyte/granulocyte cells. Importantly, this finding could be explained by the relationship between granulocytes and T cells, where cancer patients with low circulatory neutrophil-lymphocyte ratios (NLR) seem to have higher SMI compared to those with elevated NLR. For more individual markers, dendritic cells (phagocytes), which can be identified as CD11b+ cells [58], have been found to be elevated in the blood of patients with high SMI [47]. CD3−CD4+ cells, which were positively correlated with muscle fiber CSA, might belong to a subtype of T cells with a low expression of surface CD3, or even, CD4+ antigen-presenting cells [58–61]. Collectively, this evidence suggests that immune cells influence muscle mass during cancer.

Consistent associations of CD8 T cells (CD3+CD4−) with histological and radiological indices of muscle mass revealed that low numbers of CD8 T cells are present in patients with low muscle mass. The associations observed here do not imply causal mechanisms; however, one could speculate on the possibility that CD8 T cells could influence muscle mass. Further exploration on the CD8A gene, suggested an inverse association of CD8 T cells with apoptosis (caspase 8), autophagy (Beclin 1), catabolic signaling (ACVR2B and ACVR1B receptors), and ubiquitin-proteasome (FBXO32, FOXO4, MUL1, and TRIM63) pathways which collectively would result in improvement or maintenance of muscle mass (Fig. 5) [1, 2, 62]. In accordance with our findings, downregulation of the CD8A T cell gene is observed in the blood of patients experiencing loss of muscle mass, when compared to non-losing patients [57]. A pilot study, evaluating several circulating CD8 T cells subsets (naïve and memory/pro-inflammatory and anti-inflammatory) in a population with mainly gastrointestinal malignancies, provided a more complex perspective on the relationships between CD8 T cell subsets and lean mass, where pro-inflammatory CD8 T cells (expressing IL-2) were negatively associated with lean mass while other phenotypes were not associated [63].

Cross-talk between muscle and immune cells is essential to restore homeostasis and promote muscle tissue repair after a catabolic event [40]. For instance, positive correlation of CD8A gene with IL-10 receptor suggests an involvement of CD8 T cells [64, 65] and other anti-inflammatory immune cells (M2 macrophages) [66, 67] in the IL-10 pathway of the skeletal muscle which involves a reduction in the expression of pro-inflammatory mediators (IL-6, IL-1α, IL-β, TNF-α, and IFN-γ) leading to the support of anabolic hormones activity and regulation of myogenesis [67–69].

Comparison of our quantifications and observations suggest that muscle immune environment from cancer patients might differ from both healthy and inflammatory muscle disease (Table 5). We did not observe characteristics of inflammatory myopathy such as immune cell clusters, muscle fiber infiltration, and perifascicular atrophy [70]. Cautious comparisons with immune cell quantifications done in other muscle groups inform that higher and variable numbers of T cells are present in patients with muscle inflammatory disease when compared to the present cancer population [7, 11, 12]. In addition, T cells and granulocyte/phagocytes (CD11b+) are scarce in healthy individuals in comparison to people with cancer or inflammatory myopathy [7, 12].

**Table 5:** Human studies reporting number of CD3+ (T cells) and CD11b+ (granulocytes/phagocytes) in muscle

Study	Subjects	Age (years)	Sample size	Muscle group	Number of CD3+/mm^2^	Number of CD11b+/mm^2^
Anoveros-Barrera et al. (current study)	Cancer population, men and women	64 (38–81)	*n* = 30	*rectus abdominis*	12.3 (1.6–33.3)	3 (0–13)
Pandya et al. [11]	Myopathy, men and women	64 (43–74)	*n* = 16	VA and TA	13.5 (0.5–963)	N/A
Dorph et al. [12]	Myopathy, men and women	58 (38–76)	*n* = 11	VA and TA	Symptomatic muscles: 58.1 (0.5–159.8) Asymptomatic muscles: 55.9 (1.1–126.8)	N/A
	Healthy volunteers, men and women	51 (47–56)	*n* = 6	VL and TA	1.8 (0.4–5.0)	
Englund et al. [7]	Myopathy, men and women.	46 (26–64)	*n* = 11	VL	3 (0–7)	1 (0–10)
	Healthy volunteers, men and women	27 (22–46)	*n* = 7	VL	1 (0–2)	0 (0–1)

Values reported as median (minimum-maximum). Myopathy: polymyositis and dermatomyositis. *VL* vastus lateralis, *TA* tibialis anterior, *N/A* not assessed

The main scientific approach of this study was based on inter-individual variations across the range of muscle mass in cancer patients. We biopsied a representative sample of patients (Fig. 1) across the entire SMI distribution reported for patients with cancers in our geographical region, and our study did not have sampling bias issues [13]. In this distribution, some patients have muscle mass comparable to the normal range expected for healthy 30-year-old individuals [20], and others have significant muscle depletion (Fig. 1 and Table 4). We acknowledge the limitations of our current cohort, which is not completely clear how the effect of advanced cancer stage or chemotherapy exposure is accounted for. It is recognized that advanced cancer is associated with intense catabolism which influences muscle mass and immune cells [71–73]; however, patients with advanced cancer, which were located among the whole SMI distribution in our study, can experience periods of muscle mass maintenance during their disease trajectory [74]. For those patients exposed to chemotherapy prior to biopsy, again found along the SMI distribution with the exception of patients with greater muscle mass, chemotherapeutic agents promote cytotoxicity and metabolic alterations [75–77]; however, long-term effects of muscle immune environment have not been extensively explored.

Whether the muscles of cancer patients are different from those of healthy controls is a legitimate question; however, the feasibility of obtaining the *rectus abdominis* from appropriately matched healthy controls is questionable. This would be limited to healthy persons having elective abdominal surgery and CT imaging for quantification of muscle mass (a relatively infrequent event). The control population would also need to be proven free of any acute or chronic conditions associated with muscle wasting. As a consequence, a very small number of studies include healthy controls [13].

Our study confirms the presence of immune cells within the muscle of cancer patients; further exploration of immune cell phenotypes (i.e., more detailed analysis of maturation and activation) and immune mediators (e.g., cytokines) is necessary to better understand the local immune cell environment in relation to the muscle mass in cancer. Evaluation of changes in immune cells, muscle mass, and disease progression over time will expand on the findings of the current research which is limited by its cross-sectional design.

## Conclusion

This is the first study to investigate the local immune environment of the skeletal muscle by identifying immune cell populations in the muscle and their relationship to muscle mass in cancer patients. The skeletal muscle immune environment of patients with cancer is comprised of different immune cells from the adaptive and innate immune response. The muscle tissue immune environment in cancer patients might differ from healthy conditions and muscle inflammatory disease. Correlations of T cells, granulocyte/phagocytes, and CD3−CD4+ cells with histological and radiological muscle mass measurements suggest a relationship between immune cells and muscle mass status. Gene correlation analysis suggests an inverse relationship of CD8 T cells with diverse components of muscle catabolic pathways which might have an impact on muscle mass preservation during cancer. These results serve to better understand the role of the local immune environment of skeletal muscle and its implications for muscle mass in cancer patients.

## Additional files

Additional file 1: **Table S1.** Primary antibody panel for immunohistochemistry. (DOCX 21 kb)

Additional file 2: **Table S2.** Secondary antibody panel for immunohistochemistry with corresponding primary antibodies. (DOCX 20 kb)

Additional file 3: **Table S3.** Patient characteristics, secondary cohort with microarray analysis in *rectus abdominis* muscle. (DOCX 23 kb)

Additional file 4: **Table S4.** Negative univariate associations (r=<-0.50) between T cell related genes and genes involved in muscle catabolic pathways of *rectus abdominis* muscle of secondary male cohort (n=69). (DOCX 27 kb)

Additional file 5: **Table S5.** Negative univariate associations between T cell related genes and genes involved in muscle catabolic pathways of *rectus abdominis* muscle of secondary female cohort (n=64). (DOCX 25 kb)

Additional file 6: **Figure S1.** Immunostaining of CD3+CD4+ (A), CD3+CD4- (B), CD3-CD4+ (C), CD11b+CD14-CD15- (D) and CD11b+CD14+CD15+ (E) cells. Immune cells pointed by the white arrow. A.1, B.1, C.1, D.1 and E.1 antibody detected by Alexa Flour® 647. A.2, B.2, C.2, D.2 and E.2 antibody detected by Alexa Flour® 568. D.3 and E.3 antibody detected by Alexa Flour® 488. A.3, B.3, C.3, D.4 and E.4 nuclear stain detected by DAPI. A.4. B.4, C.4, D.5 and E.5 Merged images. Scale bar 45 μm. (DOCX 1768 kb)

Additional file 7: **Figure S2.** Immunostaining of serial cross-sections of muscle tissue: CD11b+CD14+CD15+ cells (A) and laminin-dystrophin (B). Stained nuclei in blue. A.1 Original image with no brightness manipulation. A.2 and A.3 Brightness was increased to visually appreciate the location of the CD11b+CD14+CD15+ cell (arrow) on the endomysial area. B. Serial cross-section used to confirm the location of immune cells on the periphery of muscle fibers (endomysium). Asterisks mark muscle fibers used as a reference point, and immune cell location is pointed by the white arrow. Scale bar 11 μm. (DOCX 1549 kb)

Additional file 8: **Figure S3.** Flow cytometry analyses done using FlowJo© software [FlowJo, LLC]. A. Gating strategy for the main cell population. B. Exclusion of doublets. C and F. Gating strategy for CD3 and CD11b positive populations. D and G. Stable flow stream for CD3 and CD11b. E and H. FMO controls for CD3 and CD11b. (PDF 443 kb)

Additional file 9: **Figure S4.** Gene arrays from *rectus abdominis* muscle from secondary female cohort (n=64). Correlation matrix of T cells genes and muscle catabolic pathway genes. Strength of the correlation is represented by the size and color intensity of each spot, positive in blue and negative in red. Pearson correlation analysis. (DOCX 1449 kb)

## Data Availability

Data for main cancer patient cohort (*n* = 30) is available upon request. Data for microarray cohort (*n* = 69) is deposited in the US National Center for Biotechnology Information (NCBI) Gene Expression Omnibus25. GEO series accession number GSE41726.

## Abbreviations

CSA: Cross-sectional area
CT: Computed tomography
HU: Hounsfield units
IL: Interleukin
L3: Lumbar 3
NFĸB: Nuclear factor kappa-B
NLR: Neutrophil lymphocyte ratio
SMI: Skeletal muscle index
TNF: Tumor necrosis factor
