# Supplementary material for: Immunohistochemical phenotyping of T cells, granulocytes, and phagocytes in the muscle of cancer patients: association with radiologically defined muscle mass and gene expression
Source: Skelet Muscle. 2019 Sep 14;9:24. doi: 10.1186/s13395-019-0209-y (PMC6744687; doi:10.1186/s13395-019-0209-y)
Supplement: Supplementary file 1 — Table S1. Primary antibody panel for immunohistochemistry. (DOCX 21 kb) [file 13395_2019_209_MOESM1_ESM.docx]

| **Table S1. Primary antibody panel for immunohistochemistry** | | | | | | | |
| --- | --- | --- | --- | --- | --- | --- | --- |
| **Primary Antibody** | **Clone** | **Species** | **Clonality** | **Dilution** | **Supplier** | | **Function** |
| CD3 | SP7 | Rabbit | Monoclonal | 1:100 | Abcam | ab16669 | T cell receptor complex |
| CD4 | IF6 | Mouse | Monoclonal | 1:100 | Abcam | ab846 | Accessory molecule for interaction with MHC class II |
| CD11b | EP1345y | Rabbit | Monoclonal | 1:200 | Abcam | ab52478 | Classical myeloid lineage marker. Integrin, leukocyte adhesion and migration |
| CD14 | 5A3 | Mouse | Monoclonal | 1:200 | ThermoFisher (Invitrogen) | MA5-14773 | Lipopolysaccharide pattern recognition co-receptor (toll like receptor) |
| CD15 | MY1 | Mouse | Monoclonal | 1:50 | ThermoFisher  (Invitrogen) | MA1-26235 | Adhesion, chemotaxis and phagocytosis |
| Laminin | - | Rabbit | Polyclonal | 1:200 | SIGMA | L9393 | Major constituents of muscle cell membranes. |
| Dystrophin | - | Rabbit | Polyclonal | 1:25 | Abcam | ab15277 |  |
