# Supplementary material for: Immunohistochemical phenotyping of T cells, granulocytes, and phagocytes in the muscle of cancer patients: association with radiologically defined muscle mass and gene expression
Source: Skelet Muscle. 2019 Sep 14;9:24. doi: 10.1186/s13395-019-0209-y (PMC6744687; doi:10.1186/s13395-019-0209-y)
Supplement: Supplementary file 2 — Table S2. Secondary antibody panel for immunohistochemistry with corresponding primary antibodies. (DOCX 20 kb) [file 13395_2019_209_MOESM2_ESM.docx]

| **Table S2. Secondary antibody panel for immunohistochemistry with corresponding primary antibodies** | | | | | | | |
| --- | --- | --- | --- | --- | --- | --- | --- |
| **Primary Antibody (Clone)** | **Secondary antibody** | **Dilution** | **Supplier** | | **Isotype** | **Species** | **Clonality** |
| CD3 (SK7) | AlexaFluor® 647 | 1:400 | Fisher | A-21245 | IgG | Rabbit | Polyclonal |
| CD4 (IF6) | AlexaFluor® 568 | 1:400 | Fisher | A-21144 | IgG1 | Mouse | Polyclonal |
| CD11b (EP1345y) | AlexaFluor® 647 | 1:400 | Fisher | A-21245 | IgG | Rabbit | Polyclonal |
| CD14 (5A3) | AlexaFluor® 568 | 1:400 | Fisher | A-21144 | IgG1 | Mouse | Polyclonal |
| CD15 (MY1) | AlexaFluor® 488 | 1:400 | Fisher | A-11008 | IgM | Mouse | Polyclonal |
| Laminin | AlexaFluor® 647 | 1:400 | Fisher | A-21245 | IgG | Rabbit | Polyclonal |
| Dystrophin |  |  |  |  | IgG |  |  |
