# Supplementary material for: Immunohistochemical phenotyping of T cells, granulocytes, and phagocytes in the muscle of cancer patients: association with radiologically defined muscle mass and gene expression
Source: Skelet Muscle. 2019 Sep 14;9:24. doi: 10.1186/s13395-019-0209-y (PMC6744687; doi:10.1186/s13395-019-0209-y)
Supplement: Supplementary file 3 — Table S3. Patient characteristics, secondary cohort with microarray analysis in rectus abdominis muscle. (DOCX 23 kb) [file 13395_2019_209_MOESM3_ESM.docx]

| **Table S3. Patient characteristics, secondary cohort with microarray analysis in rectus abdominis muscle** | | |
| --- | --- | --- |
|  | **Males (n=69)** | **Females (n=64)** |
| **Age, mean years ± SD (Min-Max)** | 59±13 (19-87) | 63±14 (21-88) |
| **Tumor type, % (n):** |  |  |
| Colorectal | 39 (27) | 14 (9) |
| Pancreas | 14 (10) | 23(15) |
| Liver or intrahepatic bile ducts | 7 (5) | 5 (3) |
| Biliary tract | 4 (3) | 8 (5) |
| Others gastrointestinal* | 9 (6) | 3 (2) |
| Others* | 8 (4) | 22 (14) |
| Non-malignant disease | 20 (14) | 25 (16) |
| **Presence of metastasis, n** | 35 | 16 |
| **Chemotherapy exposure within 3 months previous to surgery, n** | 20 | 11 |
| **BMI (kg/m^2^), mean ± SD** | 27±4 | 28±7 |
| **BMI classification**, n:** |  |  |
| Underweight | 0 | 1 |
| Normal | 23 | 16 |
| Overweight | 28 | 14 |
| Obesity I | 9 | 6 |
| Obesity II | 3 | 8 |
| Obesity III | 0 | 2 |
| **Comorbidities, n:** |  |  |
| Diabetes type II | 6 (4) | 9 (6) |
| Hypertension | 9 (6) | 16 (10) |
| CVD | 9 (6) | 5 (3) |
| Dyslipidemia | 1 (1) | 0 (0) |
| **Computed tomography body composition analysis**, mean ± SD:** |  |  |
| L3 Muscle CSA (cm^2^) | 161.8±28.3 | 135.8±37.3 |
| SMI (cm^2^/m^2^) | 52.9±7.8 | 46.8±9.5 |
| SMI z-score (SD) | 0.0±0.9 | 0.0±1.0 |
| L3 Muscle radiodensity (HU) | 36.4±9.3 | 34.7±9.4 |
| L3 VAT CSA (cm^2^) | 175.9±111.3 | 138.1±100.5 |
| *Others gastrointestinal: appendix, stomach and small intestine. *Others: gallbladder, skin, uterus, ovary, soft tissue, kidney, head and neck. **BMI: Males n=63 / Females n=47 **Computed tomography analysis: Males n=50 / Females=42. BMI, Body Mass Index; CVD, Cardiovascular disease; L3, Lumbar 3; CSA, Cross-sectional area; SMI, Skeletal muscle index; HU, Hounsfield Unit; VAT, Visceral adipose tissue; SAT, Subcutaneous adipose tissue; TAT, Total adipose tissue; SD, Standard deviation. Population has been previously described by Stretch et.al. *PLOS One* 2013 [DOI: 10.1371/journal.pone.0065380]. | | |
