# Supplementary material for: Immunohistochemical phenotyping of T cells, granulocytes, and phagocytes in the muscle of cancer patients: association with radiologically defined muscle mass and gene expression
Source: Skelet Muscle. 2019 Sep 14;9:24. doi: 10.1186/s13395-019-0209-y (PMC6744687; doi:10.1186/s13395-019-0209-y)
Supplement: Supplementary file 4 — Table S4. Negative univariate associations (r=<-0.50) between T cell related genes and genes involved in muscle catabolic pathways of rectus abdominis muscle of secondary male cohort (n=69). (DOCX 27 kb) [file 13395_2019_209_MOESM4_ESM.docx]

| **Table S4. Negative univariate associations (r=<-0.50) between T cell related genes and genes involved in muscle catabolic pathways of rectus abdominis muscle of secondary male cohort (n=69)** | | | | | |
| --- | --- | --- | --- | --- | --- |
| **T cell category** | **Gene name** | **Muscle catabolic pathway** | **Gene name** | **r** | **p** |
| T cell function | CD3G | Ubiquitin proteasome | FOXO4 | -0.59 | 1.20E-07 |
|  |  | Ubiquitin proteasome | UBE2R2 | -0.58 | 2.16E-07 |
|  | LCK | Ubiquitin proteasome | STUB1 | -0.53 | 2.24E-06 |
|  |  | Ubiquitin proteasome | UBE2B | -0.53 | 3.14E-06 |
|  |  | Ubiquitin proteasome | FOXO4 | -0.51 | 8.55E-06 |
|  | HAVCR2 | Ubiquitin proteasome | UBE2R2 | -0.55 | 1.23479E-06 |
|  |  | Ubiquitin proteasome | FOXO4 | -0.52 | 5.21839E-06 |
|  | PDCD1 | Ubiquitin proteasome | UBE2B | -0.56 | 5.93E-07 |
|  | CD28 | Ubiquitin proteasome | FBXO32 | -0.61 | 2.33E-08 |
|  |  | Ubiquitin proteasome | USP25 | -0.59 | 9.54E-08 |
|  |  | Ubiquitin proteasome | UBE2G1 | -0.57 | 2.47E-07 |
|  |  | Ubiquitin proteasome | UBR2 | -0.57 | 3.58E-07 |
|  |  | Ubiquitin proteasome | UBE2R2 | -0.54 | 1.31E-06 |
|  |  | Ubiquitin proteasome | STUB1 | -0.52 | 4.40E-06 |
|  |  | Ubiquitin proteasome | FOXO4 | -0.52 | 5.36E-06 |
|  |  | Ubiquitin proteasome | UBE2B | -0.51 | 9.54E-06 |
|  |  | Signaling | ACVR2B | -0.55 | 1.26E-06 |
|  |  | Autophagy | ATG13 | -0.52 | 3.99E-06 |
|  |  | Apoptosis | SIVA1 | -0.53 | 2.18E-06 |
|  | STAT4 | Ubiquitin proteasome | STUB1 | -0.66 | 8.60E-10 |
|  |  | Ubiquitin proteasome | PSMA7 | -0.63 | 5.49E-09 |
|  |  | Ubiquitin proteasome | USP4 | -0.62 | 1.84E-08 |
|  |  | Ubiquitin proteasome | MUL1 | -0.58 | 1.80E-07 |
|  |  | Ubiquitin proteasome | UBE2R2 | -0.57 | 2.64E-07 |
|  |  | Ubiquitin proteasome | UBE2B | -0.56 | 5.32E-07 |
|  |  | Ubiquitin proteasome | UBE2L3 | -0.55 | 1.03E-06 |
|  |  | Ubiquitin proteasome | UBB | -0.55 | 1.13E-06 |
|  |  | Ubiquitin proteasome | UBA52 | -0.55 | 1.16E-06 |
|  |  | Ubiquitin proteasome | FBXO32 | -0.55 | 1.23E-06 |
|  |  | Ubiquitin proteasome | FBXO32 | -0.55 | 1.23E-06 |
|  |  | Ubiquitin proteasome | TRIM63 | -0.54 | 1.37E-06 |
|  |  | Ubiquitin proteasome | UBA52 | -0.54 | 1.82E-06 |
|  |  | Ubiquitin proteasome | FOXO4 | -0.53 | 2.53E-06 |
|  |  | Ubiquitin proteasome | DNAJC11 | -0.52 | 3.72E-06 |
|  |  | Ubiquitin proteasome | UBC | -0.52 | 4.84E-06 |
|  |  | Ubiquitin proteasome | UBC | -0.52 | 5.67E-06 |
|  |  | Ubiquitin proteasome | FOXO4 | -0.50 | 1.02E-05 |
|  |  | Signaling | ACVR1B | -0.55 | 1.21E-06 |
|  |  | Signaling | ACVR2B | -0.53 | 2.72E-06 |
|  |  | Apoptosis/Autophagy | BECN1 | -0.61 | 2.28E-08 |
|  |  | Apoptosis/Autophagy | BECN1 | -0.58 | 2.21E-07 |
|  |  | Apoptosis | SIVA1 | -0.62 | 1.29E-08 |
|  | CD2 | Ubiquitin proteasome | FOXO4 | -0.52 | 4.96E-06 |
|  |  | Ubiquitin proteasome | STUB1 | -0.63 | 4.94E-09 |
|  |  | Ubiquitin proteasome | UBE2V1 | -0.59 | 1.00E-07 |
|  |  | Ubiquitin proteasome | UBE2R2 | -0.55 | 1.19E-06 |
|  |  | Apoptosis/Autophagy | BECN1 | -0.55 | 7.62E-07 |
|  | CD6 | Ubiquitin proteasome | FOXO4 | -0.50 | 1.15E-05 |
|  |  | Ubiquitin proteasome | STUB1 | -0.50 | 1.18E-05 |
|  | PTPRC (CD45) | Ubiquitin proteasome | STUB1 | -0.58 | 1.98E-07 |
|  |  | Ubiquitin proteasome | UBC | -0.55 | 1.19E-06 |
|  |  | Ubiquitin proteasome | UBA52 | -0.54 | 1.30E-06 |
|  |  | Ubiquitin proteasome | UBB | -0.54 | 1.65E-06 |
|  |  | Ubiquitin proteasome | DNAJC11 | -0.54 | 1.97E-06 |
|  |  | Ubiquitin proteasome | UBC | -0.53 | 2.56E-06 |
|  |  | Ubiquitin proteasome | UBC | -0.53 | 2.95E-06 |
|  |  | Ubiquitin proteasome | MUL1 | -0.52 | 4.09E-06 |
|  |  | Ubiquitin proteasome | FBXO32 | -0.52 | 5.02E-06 |
|  |  | Ubiquitin proteasome | FBXO32 | -0.51 | 9.04E-06 |
|  |  | Signaling | ACVR2B | -0.53 | 3.52E-06 |
|  |  | Signaling | ACVR2B | -0.50 | 1.18E-05 |
|  |  | Apoptosis | SIVA1 | -0.52 | 5.09E-06 |
|  | IL2RB | Ubiquitin proteasome | UBB | -0.58 | 2.04E-07 |
|  |  | Ubiquitin proteasome | UBC | -0.57 | 4.00E-07 |
|  |  | Ubiquitin proteasome | UBC | -0.56 | 4.63E-07 |
|  |  | Ubiquitin proteasome | STUB1 | -0.56 | 4.87E-07 |
|  |  | Ubiquitin proteasome | DNAJC11 | -0.56 | 4.93E-07 |
|  |  | Ubiquitin proteasome | UBA52 | -0.55 | 7.95E-07 |
|  |  | Ubiquitin proteasome | MUL1 | -0.55 | 8.43E-07 |
|  |  | Ubiquitin proteasome | UBC | -0.54 | 1.35E-06 |
|  |  | Ubiquitin proteasome | UBC | -0.54 | 1.49E-06 |
|  |  | Ubiquitin proteasome | UBE2L3 | -0.54 | 1.61E-06 |
|  |  | Ubiquitin proteasome | UBB | -0.52 | 5.27E-06 |
|  |  | Ubiquitin proteasome | FBXO32 | -0.51 | 6.77E-06 |
|  |  | Ubiquitin proteasome | UBE2B | -0.51 | 8.55E-06 |
|  |  | Signaling | ACVR2B | -0.52 | 5.83E-06 |
|  |  | Signaling | ACVR2B | -0.51 | 6.22E-06 |
|  |  | Apoptosis/Autophagy | BECN1 | -0.51 | 8.78E-06 |
| CD8 T cell specific function | FASLG | Ubiquitin proteasome | STUB1 | -0.58 | 1.32E-07 |
|  |  | Ubiquitin proteasome | USP2 | -0.53 | 3.62E-06 |
|  |  | Ubiquitin proteasome | FOXO4 | -0.52 | 4.58E-06 |
|  |  | Apoptosis/Autophagy | BECN1 | -0.54 | 1.79E-06 |
|  | GZMA | Ubiquitin proteasome | FOXO4 | -0.50 | 1.07E-05 |
|  |  | Ubiquitin proteasome | STUB1 | -0.50 | 1.18E-05 |
|  |  | Apoptosis | CASP8 | -0.51 | 7.66E-06 |
|  | GZMK | Ubiquitin proteasome | FOXO4 | -0.59 | 8.30E-08 |
|  |  | Ubiquitin proteasome | UBE2R2 | -0.58 | 1.54E-07 |
|  |  | Ubiquitin proteasome | STUB1 | -0.53 | 2.35E-06 |
|  |  | Apoptosis/Autophagy | BECN1 | -0.52 | 5.35E-06 |
| r= Pearson's correlation coefficient. p = <0.05: statistical significance. | | | | | |
