# Supplementary material for: Immunohistochemical phenotyping of T cells, granulocytes, and phagocytes in the muscle of cancer patients: association with radiologically defined muscle mass and gene expression
Source: Skelet Muscle. 2019 Sep 14;9:24. doi: 10.1186/s13395-019-0209-y (PMC6744687; doi:10.1186/s13395-019-0209-y)
Supplement: Supplementary file 5 — Table S5. Negative univariate associations between T cell related genes and genes involved in muscle catabolic pathways of rectus abdominis muscle of secondary female cohort (n=64). (DOCX 25 kb) [file 13395_2019_209_MOESM5_ESM.docx]

| **Table S5. Negative univariate associations between T cell related genes and genes involved in muscle catabolic pathways of rectus abdominis muscle of secondary female cohort (n=64)** | | | | | |
| --- | --- | --- | --- | --- | --- |
| **T cell category** | **Gene name** | **Muscle catabolic pathway** | **Gene name** | **r** | **p** |
| T cell function | CD3G | Ubiquitin proteasome | UBR2 | -0.30 | 1.76E-02 |
|  |  | Ubiquitin proteasome | FOXO4 | -0.28 | 2.47E-02 |
|  | HAVCR2 | Apoptosis | BECN1 | -0.30 | 1.80E-02 |
|  |  | Ubiquitin proteasome | UBE2L3 | -0.32 | 1.02E-02 |
|  |  | Ubiquitin proteasome | TRIM63 | -0.30 | 1.55E-02 |
|  |  | Ubiquitin proteasome | UBR2 | -0.28 | 2.40E-02 |
|  | PDCD1 | Ubiquitin proteasome | UBAP2 | -0.61 | 1.04E-07 |
|  |  | Ubiquitin proteasome | UBR3 | -0.50 | 2.31E-05 |
|  |  | Ubiquitin proteasome | FOXO4 | -0.42 | 4.93E-04 |
|  |  | Ubiquitin proteasome | UBR2 | -0.35 | 4.02E-03 |
|  |  | Ubiquitin proteasome | FOXO4 | -0.32 | 1.05E-02 |
|  |  | Ubiquitin proteasome | USP25 | -0.31 | 1.21E-02 |
|  |  | Ubiquitin proteasome | UBE2R2 | -0.31 | 1.38E-02 |
|  |  | Ubiquitin proteasome | UBE2V1 | -0.30 | 1.68E-02 |
|  |  | Ubiquitin proteasome | UBE2V1 | -0.25 | 4.93E-02 |
|  | CD28 | Apoptosis | SIVA1 | -0.37 | 2.32E-03 |
|  |  | Apoptosis | BECN1 | -0.36 | 3.91E-03 |
|  |  | Signaling | ACVR2B | -0.39 | 1.42E-03 |
|  |  | Signaling | ACVR1B | -0.27 | 2.79E-02 |
|  |  | Ubiquitin proteasome | UBC | -0.37 | 2.59E-03 |
|  |  | Ubiquitin proteasome | PSMA7 | -0.36 | 3.67E-03 |
|  |  | Ubiquitin proteasome | UBE2V1 | -0.35 | 4.15E-03 |
|  |  | Ubiquitin proteasome | FOXO4 | -0.33 | 8.25E-03 |
|  |  | Ubiquitin proteasome | UBC | -0.29 | 1.80E-02 |
|  |  | Ubiquitin proteasome | UBE2B | -0.29 | 1.88E-02 |
|  |  | Ubiquitin proteasome | UBA52 | -0.29 | 2.07E-02 |
|  |  | Ubiquitin proteasome | UBB | -0.27 | 2.82E-02 |
|  |  | Ubiquitin proteasome | TRIM63 | -0.26 | 4.03E-02 |
|  |  | Ubiquitin proteasome | UBR2 | -0.25 | 4.28E-02 |
|  |  | Ubiquitin proteasome | UBC | -0.25 | 4.75E-02 |
|  | STAT4 | Ubiquitin proteasome | PSMA7 | -0.28 | 2.56E-02 |
|  | CD2 | Ubiquitin proteasome | USP2 | -0.37 | 2.38E-03 |
|  |  | Ubiquitin proteasome | STUB1 | -0.33 | 7.25E-03 |
|  |  | Ubiquitin proteasome | UBE2V1 | -0.32 | 1.09E-02 |
|  |  | Ubiquitin proteasome | UBE2V1 | -0.28 | 2.55E-02 |
|  |  | Ubiquitin proteasome | FOXO4 | -0.28 | 2.78E-02 |
|  | CD6 | Ubiquitin proteasome | FOXO4 | -0.29 | 1.88E-02 |
|  | PTPRC (CD45) | Apoptosis | BECN1 | -0.30 | 1.65E-02 |
|  |  |  |  |  |  |
|  |  | Apoptosis | SIVA1 | -0.27 | 3.07E-02 |
|  |  | Signaling | ACVR2B | -0.47 | 9.17E-05 |
|  |  | Signaling | ACVR1B | -0.41 | 7.61E-04 |
|  |  | Ubiquitin proteasome | STUB1 | -0.55 | 2.10E-06 |
|  |  | Ubiquitin proteasome | UBB | -0.51 | 2.00E-05 |
|  |  | Ubiquitin proteasome | UBA52 | -0.50 | 3.16E-05 |
|  |  | Ubiquitin proteasome | UBC | -0.49 | 3.62E-05 |
|  |  | Ubiquitin proteasome | UBC | -0.48 | 5.33E-05 |
|  |  | Ubiquitin proteasome | UBB | -0.48 | 5.70E-05 |
|  |  | Ubiquitin proteasome | UBC | -0.45 | 2.25E-04 |
|  |  | Ubiquitin proteasome | UBC | -0.44 | 2.34E-04 |
|  |  | Ubiquitin proteasome | UBA52 | -0.41 | 8.11E-04 |
|  |  | Ubiquitin proteasome | UBE2R2 | -0.37 | 2.48E-03 |
|  |  | Ubiquitin proteasome | UBA52 | -0.36 | 3.60E-03 |
|  |  | Ubiquitin proteasome | PSMA7 | -0.35 | 4.17E-03 |
|  |  | Ubiquitin proteasome | USP2 | -0.32 | 9.14E-03 |
|  |  | Ubiquitin proteasome | DNAJC11 | -0.30 | 1.49E-02 |
|  |  | Ubiquitin proteasome | TRIM63 | -0.26 | 3.67E-02 |
|  |  | Ubiquitin proteasome | MUL1 | -0.27 | 3.29E-02 |
|  |  | Ubiquitin proteasome | FOXO4 | -0.26 | 3.68E-02 |
|  |  | Ubiquitin proteasome | UBE2V1 | -0.26 | 4.09E-02 |
|  | IL2RB | Apoptosis | BECN1 | -0.31 | 1.39E-02 |
|  | IL2RB | Signaling | ACVR2B | -0.33 | 8.24E-03 |
|  | IL2RB | Ubiquitin proteasome | UBC | -0.46 | 1.36E-04 |
|  | IL2RB | Ubiquitin proteasome | UBC | -0.44 | 2.58E-04 |
|  | IL2RB | Ubiquitin proteasome | UBC | -0.40 | 9.27E-04 |
|  | IL2RB | Ubiquitin proteasome | FOXO4 | -0.32 | 9.18E-03 |
| CD8 T cell specific function | FASLG | Ubiquitin proteasome | USP2 | -0.40 | 1.09E-03 |
|  | FASLG | Ubiquitin proteasome | FOXO4 | -0.30 | 1.50E-02 |
|  | GZMA | Ubiquitin proteasome | USP2 | -0.35 | 4.75E-03 |
|  | GZMK | Ubiquitin proteasome | USP2 | -0.33 | 6.89E-03 |
|  | GZMK | Ubiquitin proteasome | STUB1 | -0.27 | 3.32E-02 |
| r= Pearson's correlation coefficient. p = <0.05: statistical significance. Genes analyzed in the preset table are based on the univariate negative gene correlations from the secondary male cohort (a 46 gene list). No significant or negative correlations where observed for LCK, CASP8, ATG13, FBXO32, UBE2L3 and USP4. | | | | | |
