# Supplementary figures and images for: Immunohistochemical phenotyping of T cells, granulocytes, and phagocytes in the muscle of cancer patients: association with radiologically defined muscle mass and gene expression
Source: Skelet Muscle. 2019 Sep 14;9:24. doi: 10.1186/s13395-019-0209-y (PMC6744687; doi:10.1186/s13395-019-0209-y)

**Data Supplements**

**Figure S1**


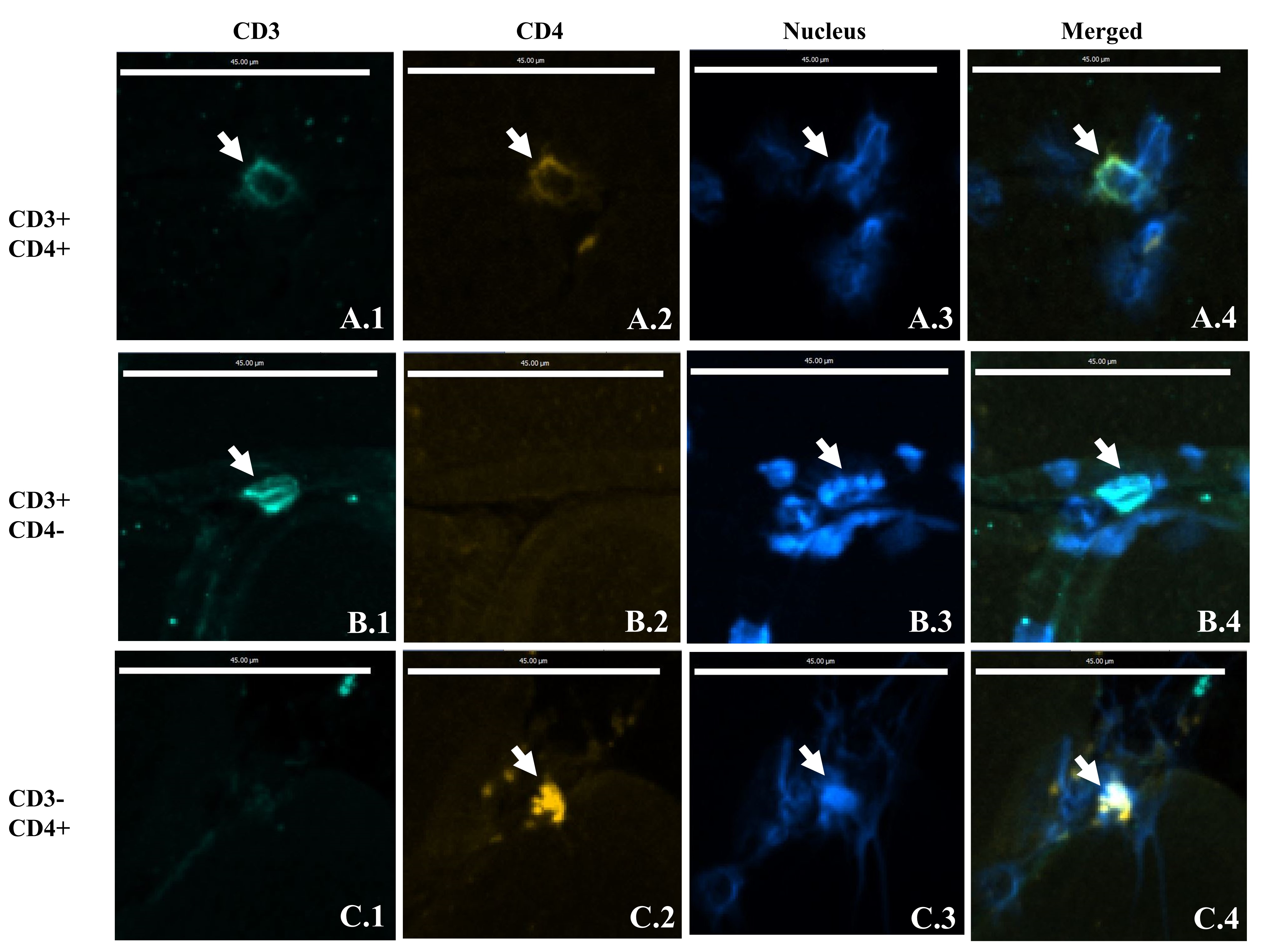

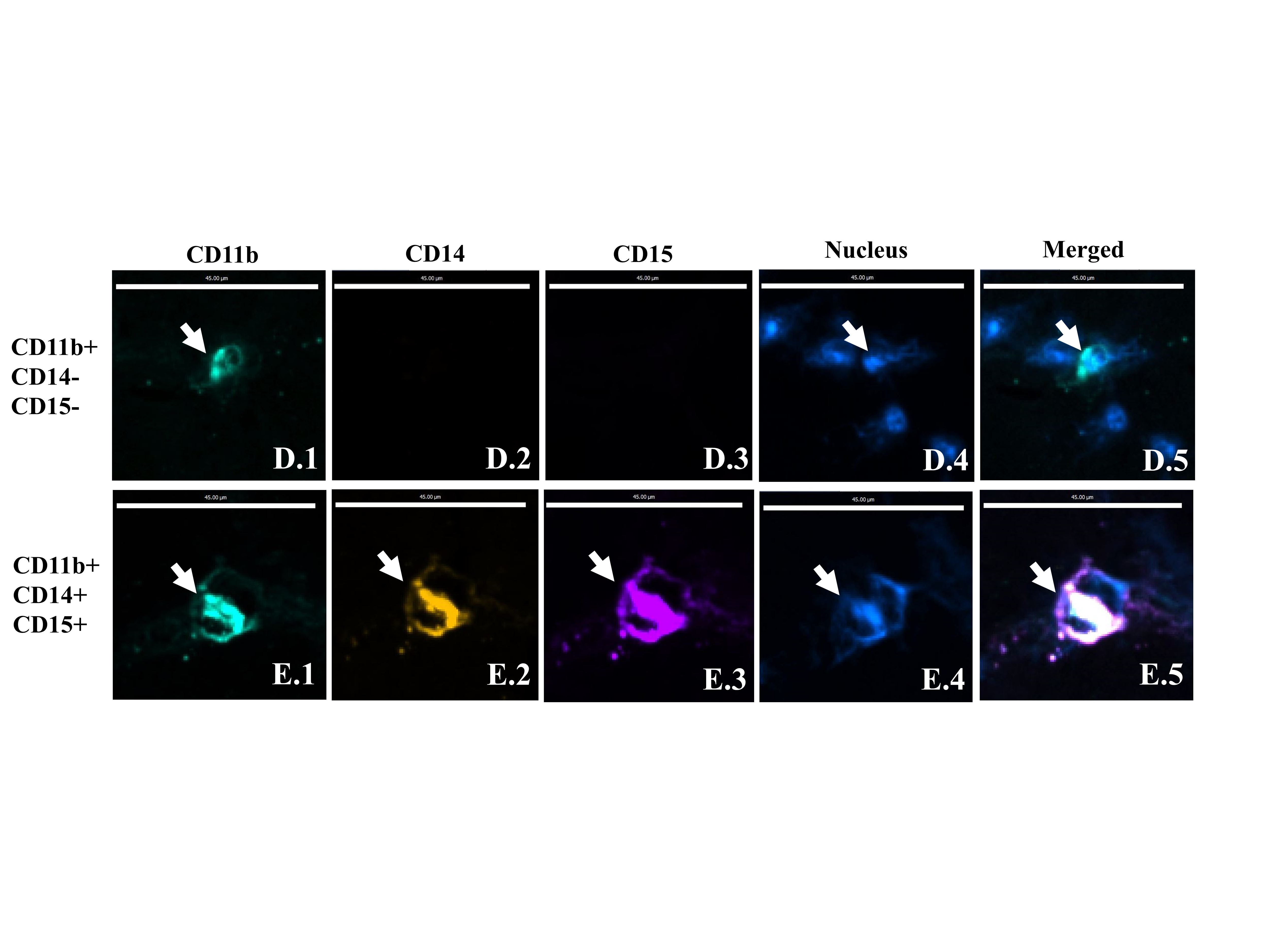

Supplement: Supplementary file 6 — Figure S1. Immunostaining of CD3+CD4+ (A), CD3+CD4- (B), CD3-CD4+ (C), CD11b+CD14-CD15- (D) and CD11b+CD14+CD15+ (E) cells. Immune cells pointed by the white arrow. A.1, B.1, C.1, D.1 and E.1 antibody detected by Alexa Flour® 647. A.2, B.2, C.2, D.2 and E.2 antibody detected by Alexa Flour® 568. D.3 and E.3 antibody detected by Alexa Flour® 488. A.3, B.3, C.3, D.4 and E.4 nuclear stain detected by DAPI. A.4. B.4, C.4, D.5 and E.5 Merged images. Scale bar 45 μm. (DOCX 1768 kb) [file 13395_2019_209_MOESM6_ESM.docx]

**Data Supplements**

**Figure S2**


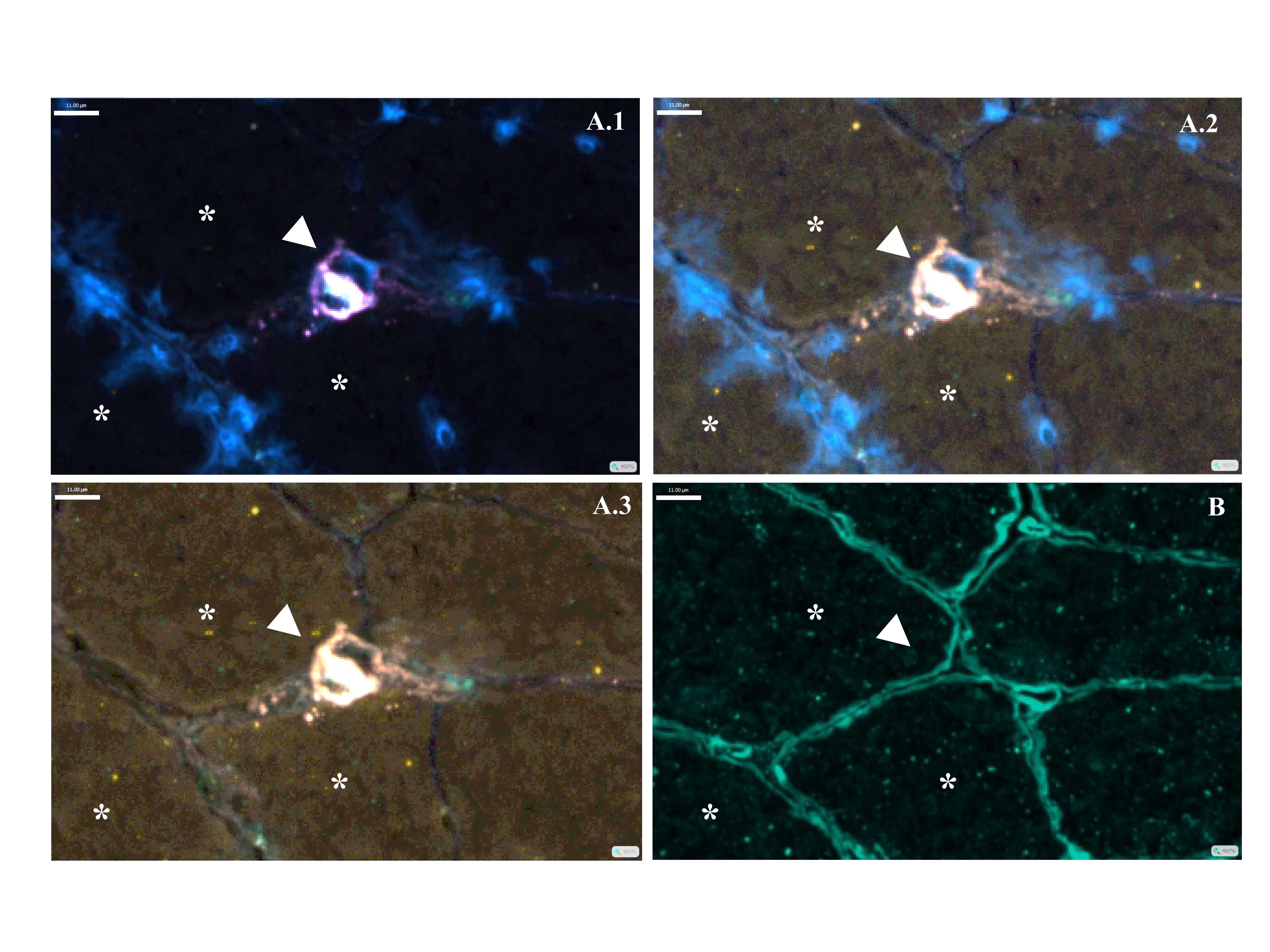

Supplement: Supplementary file 7 — Figure S2. Immunostaining of serial cross-sections of muscle tissue: CD11b+CD14+CD15+ cells (A) and laminin-dystrophin (B). Stained nuclei in blue. A.1 Original image with no brightness manipulation. A.2 and A.3 Brightness was increased to visually appreciate the location of the CD11b+CD14+CD15+ cell (arrow) on the endomysial area. B. Serial cross-section used to confirm the location of immune cells on the periphery of muscle fibers (endomysium). Asterisks mark muscle fibers used as a reference point, and immune cell location is pointed by the white arrow. Scale bar 11 μm. (DOCX 1549 kb) [file 13395_2019_209_MOESM7_ESM.docx]

## Data Supplements

Figure S3

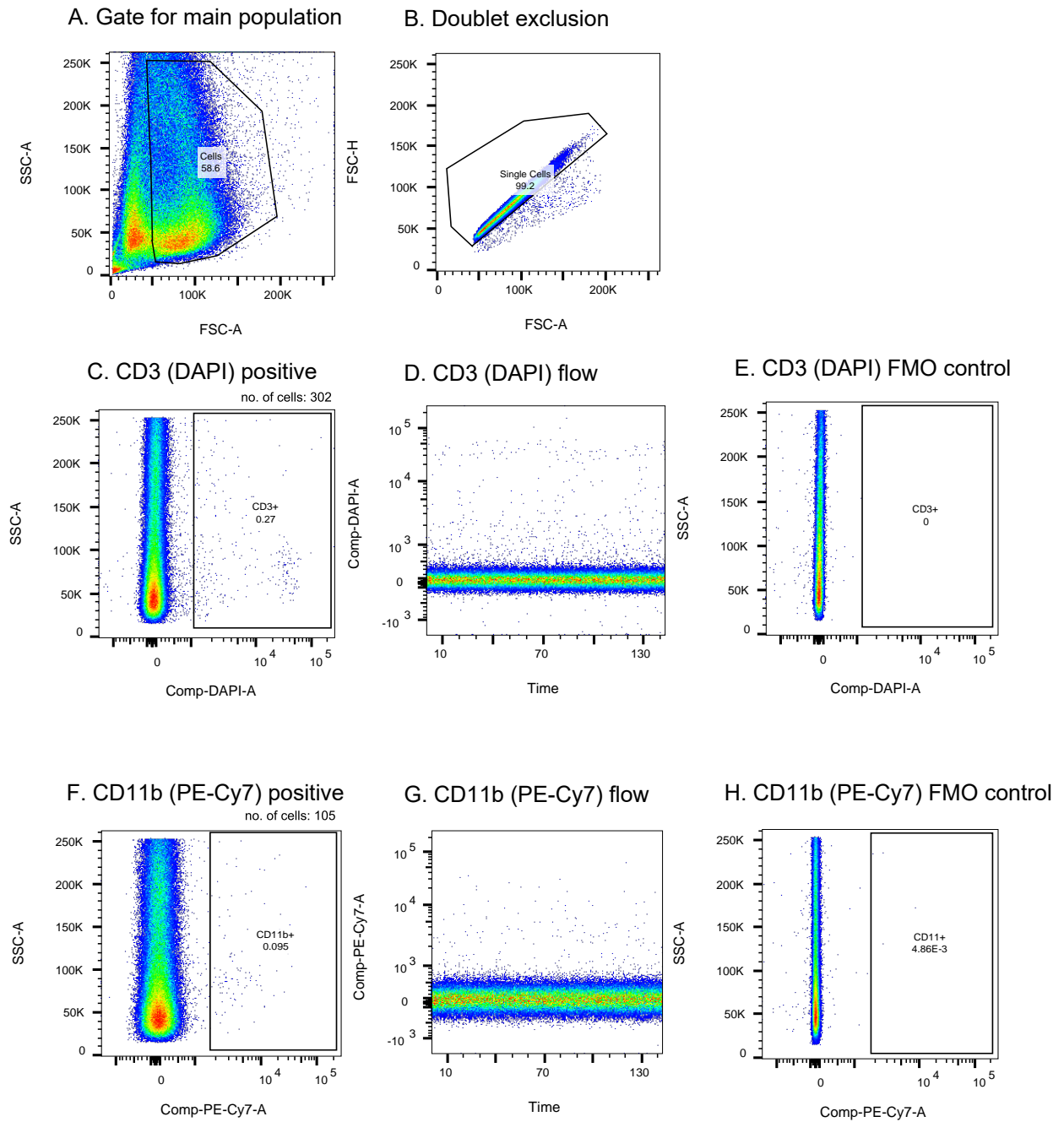

FMO: Fluorescence minus one control

Supplement: Supplementary file 8 — Figure S3. Flow cytometry analyses done using FlowJo© software [FlowJo, LLC]. A. Gating strategy for the main cell population. B. Exclusion of doublets. C and F. Gating strategy for CD3 and CD11b positive populations. D and G. Stable flow stream for CD3 and CD11b. E and H. FMO controls for CD3 and CD11b. (PDF 443 kb) [file 13395_2019_209_MOESM8_ESM.pdf]

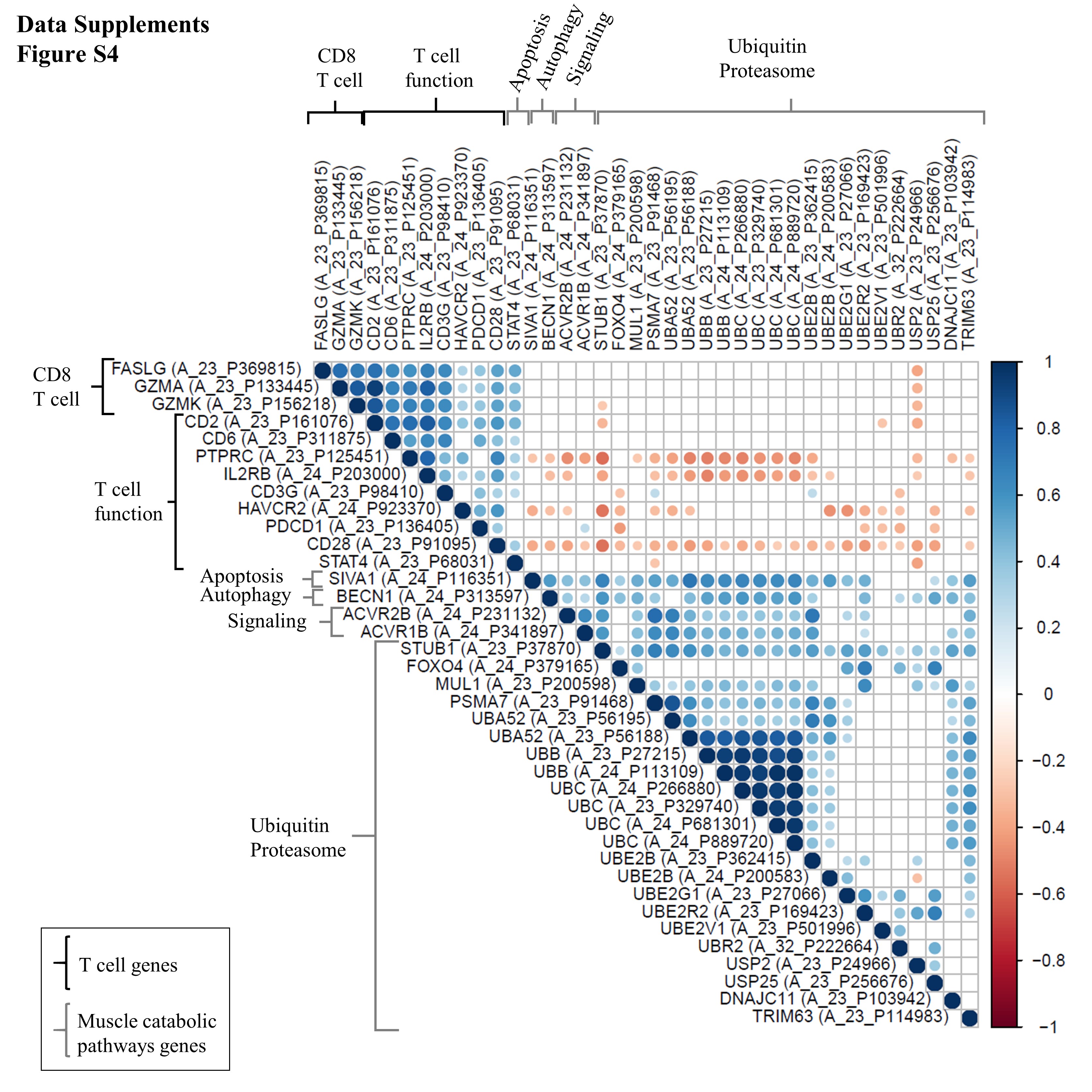

Supplement: Supplementary file 9 — Figure S4. Gene arrays from rectus abdominis muscle from secondary female cohort (n=64). Correlation matrix of T cells genes and muscle catabolic pathway genes. Strength of the correlation is represented by the size and color intensity of each spot, positive in blue and negative in red. Pearson correlation analysis. (DOCX 1449 kb) [file 13395_2019_209_MOESM9_ESM.docx]
